# S-nitrosylation of AMPKγ impairs coronary collateral circulation and disrupts VSMC reprogramming

**DOI:** 10.1038/s44319-023-00015-3

**Published:** 2023-12-18

**Authors:** Wenwu Bai, Tao Guo, Han Wang, Bin Li, Quan Sun, Wanzhou Wu, Jiaxiong Zhang, Jipeng Zhou, Jingmin Luo, Moli Zhu, Junxiu Lu, Peng Li, Bo Dong, Shufang Han, Xinyan Pang, Guogang Zhang, Yongping Bai, Shuangxi Wang

**Affiliations:** 1 National Key Laboratory for Innovation and Transformation of Luobing Theory; The Key Laboratory of Cardiovascular Remodeling and Function Research, Chinese Ministry of Education, Chinese National Health Commission and Chinese Academy of Medical Sciences; Department of Cardiology, Qilu Hospital of Shandong University, Jinan, China; 2 Department of Cardiology, Central Hospital Affiliated to Shandong First Medical University, Jinan, Shandong China; 3 Department of Geriatric Medicine and Coronary Circulation Center, National Clinical Research Center for Geriatric Disorders, Xiangya Hospital, Central South University, Changsha, Hunan China; 4 School of Pharmacy, Henan International Joint Laboratory of Cardiovascular Remodeling and Drug Intervention, Xinxiang Medical University, Xinxiang, Henan China; 5 Department of Cardiology, Shandong Provincial Hospital, Jinan, Shandong China; 6 Department of Cardiology, The 960th Hospital of PLA Joint Logistics Support Force, Jinan, China; 7 Department of Cardiovascular Surgery, The Second Hospital of Shandong University, Jinan, Shandong China

**Keywords:** Nitrosative Stress, Collateral Circulation, AMP-Activated Protein Kinase, Vascular Smooth Muscle Cells, Phenotypic Restoration, Cardiovascular System, Post-translational Modifications & Proteolysis, Vascular Biology & Angiogenesis

## Abstract

Collateral circulation is essential for blood resupply to the ischemic heart, which is dictated by the contractile phenotypic restoration of vascular smooth muscle cells (VSMC). Here we investigate whether S-nitrosylation of AMP-activated protein kinase (AMPK), a key regulator of the VSMC phenotype, impairs collateral circulation. In rats with collateral growth and development, nitroglycerin decreases coronary collateral blood flow (CCBF), inhibits vascular contractile phenotypic restoration, and increases myocardial infarct size, accompanied by reduced AMPK activity in the collateral zone. Nitric oxide (NO) S-nitrosylates human recombinant AMPKγ1 at cysteine 131 and decreases AMP sensitivity of AMPK. In VSMCs, exogenous expression of S-nitrosylation-resistant AMPKγ1 or deficient NO synthase (iNOS) prevents the disruption of VSMC reprogramming. Finally, hyperhomocysteinemia or hyperglycemia increases AMPKγ1 S-nitrosylation, prevents vascular contractile phenotypic restoration, reduces CCBF, and increases the infarct size of the heart in *Apoe*^*-/-*^ mice, all of which is rescued in *Apoe*^*-/-*^*/iNOS*^*sm-/-*^ mice or *Apoe*^*-/-*^ mice with enforced expression of the AMPKγ1-C130A mutant following RI/MI. We conclude that nitrosative stress disrupts coronary collateral circulation during hyperhomocysteinemia or hyperglycemia through AMPK S-nitrosylation.

## Synopsis

Nitrosative stress impairs coronary collateral circulation during metabolic stress. It induces iNOS-mediated AMPK S-nitrosylation to disrupt VSMC reprogramming.

- Nitrosative stress is a novel mechanism of poor coronary collateral circulation under hyperhomocysteinemia or hyperglycemia.
- AMPK activity is regulated by iNOS-NO-directed AMPKγ S-nitrosylation.
- Targeting VSMC reprogramming might be an adjuvant therapy or a substitute of the surgery-based reperfusion therapy.

## Abstract (web summary)

Nitrosative stress impairs coronary collateral circulation during metabolic stress. It induces iNOS-mediated AMPK S-nitrosylation to disrupt VSMC reprogramming.

## Graphical abstract

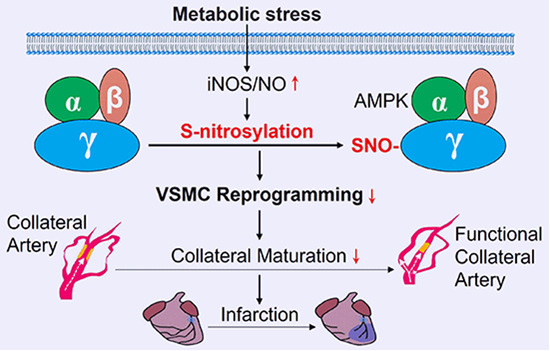

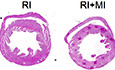

## Introduction

A rapid blood flow resupply through coronary collateral circulation is vital to preserve ischemic heart in patients with acute myocardial infarction (MI) (Seiler, 2010). Patients with diabetes and metabolic syndrome not only have a higher risk of ischemic heart diseases, but also have little to no coronary collateral circulation, causing the worse clinical outcome of acute MI (Guo et al, 2021; Li et al, 2019b; Regieli et al, 2009). The molecular mechanism how coronary collateral circulation is impaired by metabolic risk factors is poorly understood, limiting the effective approaches to treat MI (Ding et al, 2020).

Generally, collaterals develop through two distinct stages including growth and maturation. In the first stage, vascular smooth muscle cell (VSMC) switches from the adult, quiescent, contractile phenotype to the synthetic, proliferative and migratory phenotype into the lumen of native collateral vessel (Das et al, 2019; Tang and Fang, 2017). Collateral artery is closed without blood flow. In the second stage, due to gradient pressure produced by arterial occlusion, these pre-existing collateral arteries are opened with increased-diameter so as to provide blood perfusion to the collateral dependent ischemic regions, in which VSMC phenotype is characteristically contractile, but not proliferative (Eitenmuller et al, 2006; Faber et al, 2014; Wang et al, 2023). Here, we thought that there is a transition between growth and maturation because VSMC should reprogram from the synthetic to the contractile prior to the opening. This step is critical to determine the formation of functional collateral artery. So far, many studies have addressed arterial growth in physical exercise and hypoxia (Aghajanian et al, 2021; Mobius-Winkler et al, 2016). However, the mechanism orchestrating VSMC reprogramming is underexplored.

Nitrosative stress, a nitric oxide (NO)-mediated nitrosylation of redox-sensitive thiols, has been linked to the regulation of signal transduction, gene expression, and cell growth and apoptosis (Chamorro et al, 2016; Yin et al, 2022). As reported recently, nitrosative stress drives heart failure with preserved ejection fraction (Schiattarella et al, 2019). Protein S-nitrosylation plays critical roles in nitrate tolerance and cardiac diastolic dysfunction (Yoon et al, 2021; Zhou et al, 2019). AMP-activated protein kinase (AMPK) is a heterodimer composed of a α-catalytic subunit and β/γ-regulatory subunits, in which the γ subunit is the key for the enzyme to sense AMP level within the cell (Li et al, 2015). We and others have demonstrated that AMPKα deficiency induces VSMC dysfunctions and phenotypic switching (Lee et al, 2016; Liang et al, 2018; Wang et al, 2012).

Given the critical role of VSMC fate reprogramming in collateral circulation, we speculated that nitrosative stress increases cardiovascular risk by attenuating the formation of coronary collateral circulation. Our studies show that hyperhomocysteinemia (HHcy) and hyperglycemia, the two important components associated with cardiac ischemia by upregulating iNOS-NO signaling (Gawrys et al, 2020), induced AMPKγ1 S-nitrosylation, which, in turn impairs VSMC contractile phenotypic restoration of the newly developed collateral artery. We showed that iNOS-mediated S-nitrosylation at C131 of AMPK reduces its activity and disrupts collateral circulation. Importantly, our results suggest that alleviation of nitrosative stress improves recovery following MI in both rat and mouse models.

## Results

### Nitroglycerin (NTG) infusion induces nitrosative stress and promotes myocardial injury in rats with repetitive ischemia (RI) plus MI

**Figure 1:**
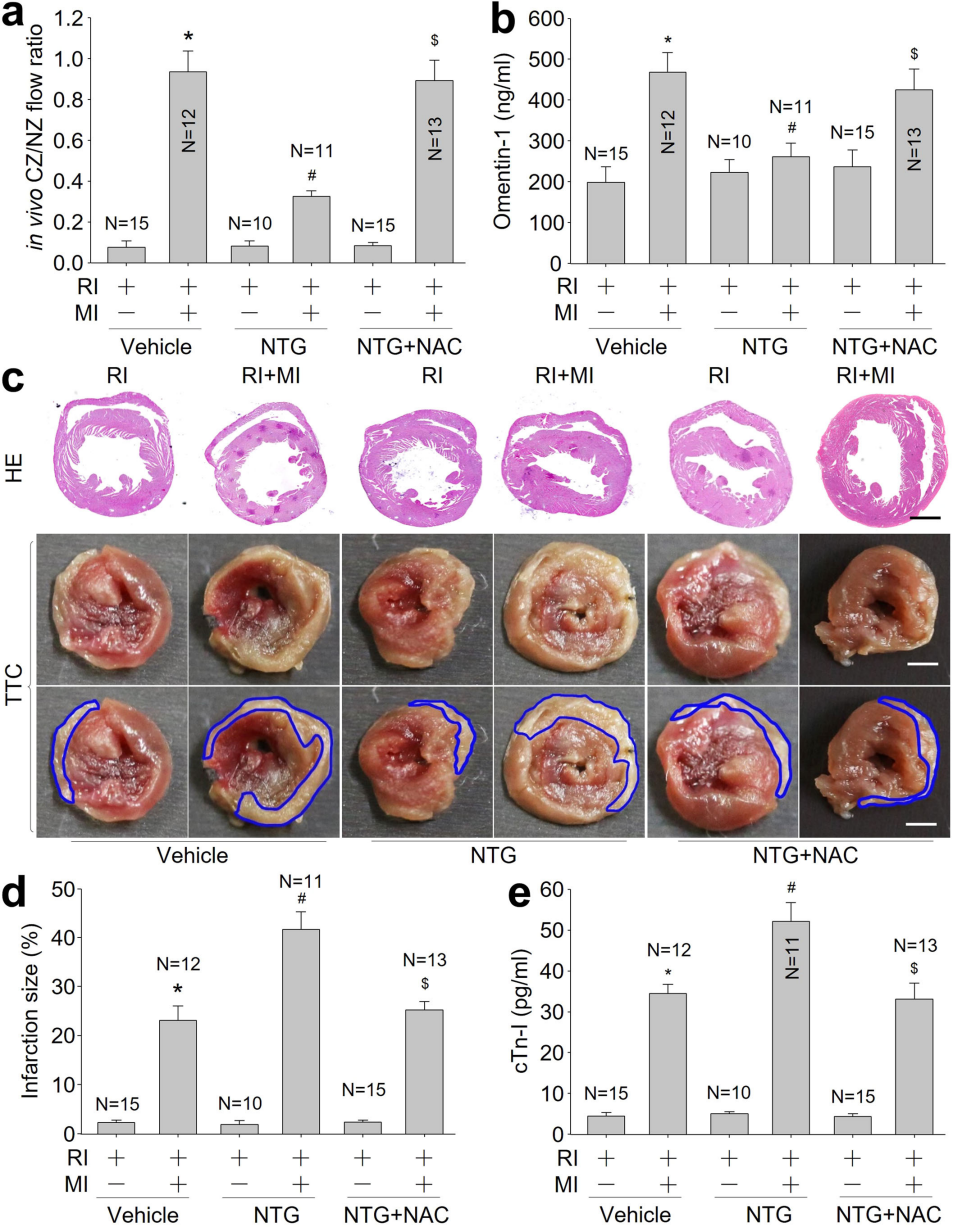
Continuous infusion of nitroglycerin (NTG) decreases coronary collateral blood flow and increases heart infarcted size in rats with RI/MI, which are abolished by *N*-acetyl-cysteine (NAC). (**A**) The animal protocol was depicted in Appendix Fig. S1a. Coronary blood flow was measured in collateral zone (CZ) and normal zone (NZ) using microspheres, and in vivo coronary collateral blood flow (CCBF) was expressed as the ratio of CZ/NZ flow. (**B**) Plasma omentin-1 level was assayed using ELISA. (**C**) The morphology of heart was determined by HE or TTC staining. (**D**) Quantitative analysis of infarction size was performed. (**E**) Plasma cTn-I level was determined using ELISA. The scale bar represents 500 µm. Error bars are mean ± SEM. *P < 0.05 vs. vehicle plus RI. ^#^*P* < 0.05 vs. vehicle plus RI/MI. ^$^*P* < 0.05 vs. NTG plus RI/MI. A one-way ANOVA followed by Tukey post hoc tests was used.

To determine the role of nitrosative stress in the formation of coronary collateral circulation, the coronary collateral growth was stimulated by transient repetitive coronary artery occlusion and nitrosative stress was induced by 5-day NTG infusion in rats (Appendix Fig. S1a). MI surgery was performed to trigger the maturation of pre-existing collateral artery due to pressure gradient (Eitenmuller et al, 2006). Consistent with our previous reports (Zhou et al, 2019), NTG infusion induced nitrosative stress as increased levels of S-nitrosylated proteins and 4-HNE (Appendix Fig. S1b–e). We also observed that acute MI increased CCBF (Fig. 1A), the levels of plasma omentin-1 (Fig. 1B), which is a predictor of good collateral circulation identified by us previously (Fang et al, 2020; Zhou et al, 2017), and ex vivo coronary flow determined by Langendorff-perfusion (Appendix Table S1). However, 5-day NTG infusion decreased coronary flow, CCBF and plasma omentin-1 level, compared to vehicle-treated rats with RI/MI. As expected, NTG increased the infarction sizes of hearts and serum cardiac troponin I (cTn-I) levels (Appendix Fig. S1c–e), and promoted cardiac dysfunctions (Appendix Table S1).

### Inhibition of S-nitrosylation by N-acetyl-cysteine (NAC) abolishes NTG-impaired coronary collateral circulation

To determine whether NO-mediated S-nitrosylation contributes to NTG-impaired coronary collateral circulation, NTG-infused rats were treated with NAC, which serves as a precursor of glutathione synthesis to block S-nitrosylation (Mani et al, 2013). As depicted in Appendix Fig. S1b–e, NAC inhibited protein S-nitrosylations and 4-HNE production in the collateral zone of ischemic heart. In NTG-infused rats with RI/MI, co-administration of NAC markedly comprised the effects of NTG on CCBF, plasma omentin-1 levels, infarction sizes, serum cTn-I levels, and cardiac dysfunctions (Fig. 1A–E and Appendix Table S1). These data reveal that nitrosative stress via S-nitrosylation disrupts the hemodynamics of coronary collateral circulation following MI (Appendix Fig. S2).

### Nitrosative stress via S-nitrosylation inhibits vascular contractile phenotypic restoration in rats with RI/MI

**Figure 2:**
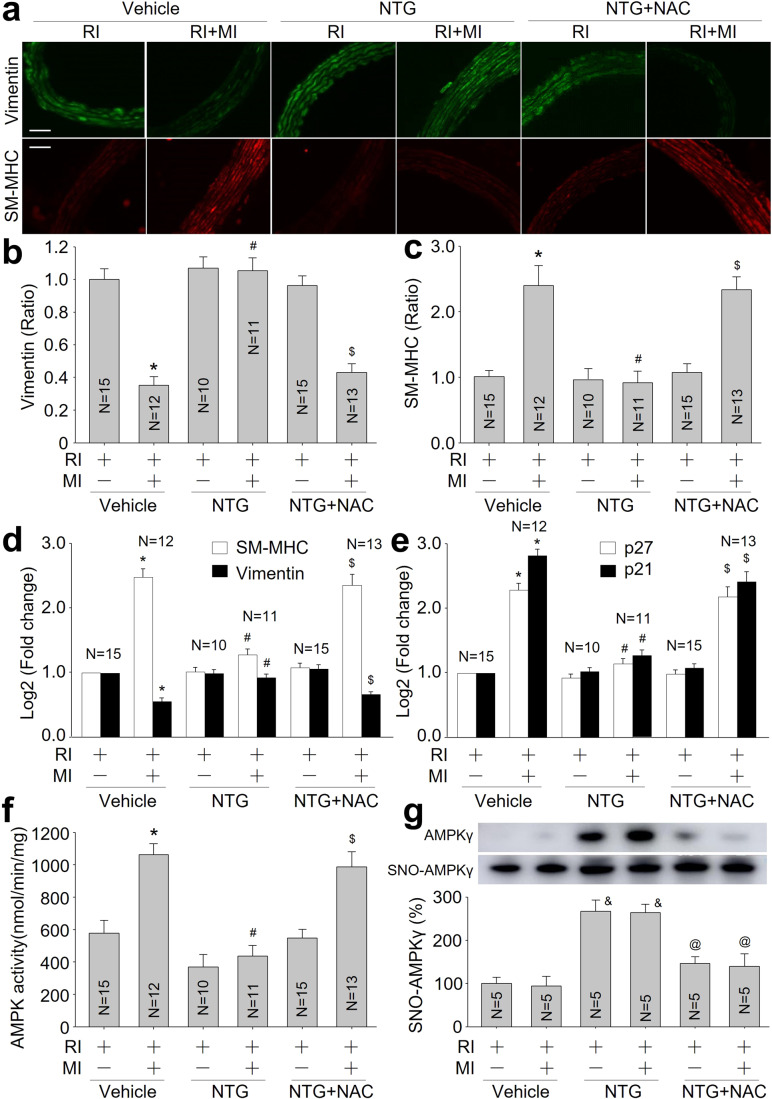
Continuous infusion of nitroglycerin (NTG) impairs vascular phenotypic restoration and AMPK activity in hearts isolated from rats with RI/MI through nitrosative stress. (**A**) The animal protocol was depicted in Appendix Fig. S1a. IFC analyses of contractile marker SM-MHC and synthetic marker vimentin in coronary artery. (**B**) Quantitative analyses of vimentin. (**C**) Quantitative analyses of SM-MHC. (**D**) Gene expressions of vimentin and SM-MHC were conducted using quantitative PCR. (**E**) Gene expression levels of p21 and p27 were conducted using quantitative PCR. (**F**) The AMPK activity in heart tissue isolated from collateral zone. (**G**) AMPKγ1 S-nitrosylation in heart tissue isolated from collateral zone. The scale bar represents 50 µm. Error bars are mean ± SEM. *P < 0.05 vs. RI plus vehicle. ^#^*P* < 0.05 vs. RI/MI plus vehicle. ^$^*P* < 0.05 vs. RI/MI plus NTG. ^&^*P* < 0.05 vs. vehicle plus RI or RI/MI. ^@^*P* < 0.05 vs. NTG plus RI or RI/MI. A one-way ANOVA followed by Tukey post hoc tests was used.

The conversion of vascular smooth muscle cell (VSMC) from contractile to synthetic phenotype, also called VSMC reprogramming in this study, is essential for the maturation of the pre-existing collateral artery (Hutcheson et al, 2013). Therefore, we examined vascular phenotypes by measuring contractile marker SM-MHC, synthetic marker vimentin, and proliferative markers (p27 and p21). As indicated in Fig. 2A–E, acute MI upregulated SM-MHC but decreased vimentin, compared to rats without MI. Gene expression levels of p27 and p21 (cell cycle inhibitors) were downregulated by NTG. These alterations induced by NTG were abolished by NAC, indicating that nitrosative stress via S-nitrosylation prevents vascular contractile phenotypic restoration.

### Nitrosative stress induces AMPKγ S-nitrosylation in vivo

As a highly aerobic organ, AMPK activation shows a protective effect on heart under ischemia (Noppe et al, 2014). As reported in Fig. 2F, compared to vehicle-treated rats, AMPK activity was reduced in the collateral zone of ischemic heart, which were abrogated by NAC co-treatment. We also measured AMPKγ S-nitrosylation using biotin-switched method and observed that NTG administration remarkably increased AMPKγ S-nitrosylation in the collateral zone, which were bypassed by NAC (Fig. 2G).

### NO directly S-nitrosylates human recombinant AMPKγ1 protein in vitro

**Figure 3:**
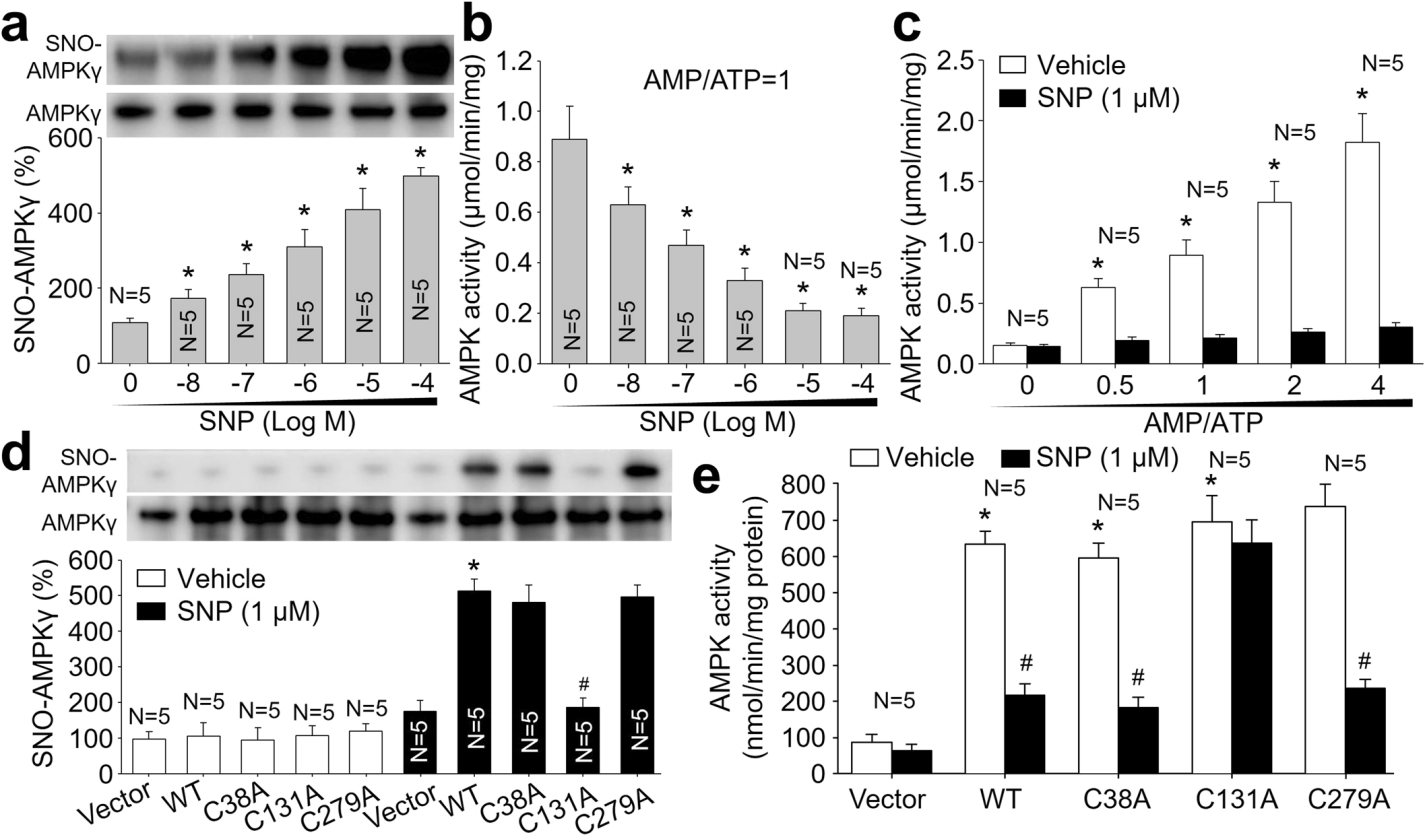
Human AMPKγ1 is S-nitrosylated at cysteine 131, which decreases the response of AMPK to AMP. (**A**) Recombinant human AMPKα1β1γ1 protein complex were incubated with sodium nitroprusside (SNP, 0.001–10 μM) for 2 h in reaction buffers. Reaction products were subjected to determine AMPKγ1 S-nitrosylation using biotin-switch method. (**B**) AMPK catalytic activity by ^32^P-SAMS peptide method under AMP = ATP. (**C**) Recombinant human AMPKα1β1γ1 protein complex was incubated SNP (1 μM) for 2 h and subjected to detect AMPK catalytic activity with the ratio of AMP/ATP at 0-4 in reaction buffers. (**D**) HEK293 cells were transfected with plasmids expressing AMPKγ1 (WT, C38A, C131A, C279A) for 48 h and then treated with SNP (1 μM) for 2 h. His-tagged AMPKγ1 protein purified from total cell lysates was subjected to measure AMPKγ1 S-nitrosylation. (**E**) AMPKα protein purified from total cell lysates was subjected to measure AMPK catalytic activity. Error bars are mean ± SEM. *P < 0.05 vs. Vehicle (point 0) in (**A**, **B**). *P < 0.05 vs. AMP/ATP at 0 in (**C**). *P < 0.05 vs. Vehicle plus WT. ^#^*P* < 0.05 vs. SNP plus WT. Repeated-measures ANOVA was used in (**A**–**C**). A one-way ANOVA followed by Tukey post hoc tests was used in (**D**, **E**).

AMPKγ1 isoform is ubiquitously expressed, while AMPKγ2/3 show the more restricted expression in skeletal muscle, heart, and placenta, but not in artery (Steinberg and Kemp, 2009). To determine whether NTG-induced AMPK inhibition is a direct effect, purified human AMPKγ1 protein was incubated with sodium nitroprusside (SNP), an NO donor (Zhou et al, 2019). As depicted in Fig. 3A, in vitro exposure of purified AMPKγ1 protein to SNP caused a dose-dependent AMPKγ1 S-nitrosylation, excluding the possibility of AMPKγ S-nitrosylation through an indirect effect of NO.

### S-nitrosylated AMPKγ1 reduces AMPK activity by desensitizing AMP

We next determined the effects of AMPKγ1 S-nitrosylation on AMPK activity by treating recombinant human AMPKα1β1γ1 protein complex with SNP and detected the catalytic activity of AMPKα with the ratio of AMP/ATP at 1 in kinase buffer as described previously (Wang et al, 2012). SNP dose-dependently inhibited AMPK catalytic activity in vitro (Fig. 3B), showing that NO-mediated AMPKγ1 S-nitrosylation suppresses AMPK activity.

As reported previously (Garcia and Shaw, 2017), AMPK restores the ATP supply by sensing the ratio of adenine nucleotides through competitive binding of AMP/ADP/ATP to cystathionine beta synthase sites in γ subunit, which varies in length but share a conserved COOH-terminal ~300 residues (Appendix Fig. S3a). We thought that AMPKγ1 S-nitrosylation suppresses AMPK activity by affecting AMPKγ1 as a sensor of AMP. To test this notion, we measured AMPK activity under the different ratios of AMP to ATP in kinase buffer. As observed in Fig. 3C, the catalytic activity of vehicle-incubated recombinant human AMPKα1β1γ1 protein was increased when the ratio of AMP to ATP was increased. However, AMPK activity was not increased if we enhanced the ratio of AMP to ATP.

### NO inhibits AMPK activity by S-nitrosylating AMPKγ1 cysteine 131

To uncover how NO S-nitrosylates AMPKγ1, we performed analyses of amino acid sequence to identify the potential sites since S-nitrosylation is an NO-directed modification of cysteine thiol (Murphy et al, 2014). As seen in Appendix Fig. S3b,c, AMPKγ1 proteins are highly conserved between human and mouse. In human AMPKγ1 protein, the 38^th^, 131^st^, and 279^th^ amino acids are cysteine (C38/C131/C279), equal to C37/C130/C278 in mouse AMPKγ1 protein. In support of our hypothesis that S-nitrosylation downregulates AMPK activity, both cysteine 38 and cysteine 279 are close, while cysteine 131 locates next to the AMP binding site (Appendix Fig. S3d). We generated plasmids expressing His-AMPKγ1-WT, His-AMPKγ1-C38A, His-AMPKγ1-C131A, and His-AMPKγ1-C279A with replacements of cysteine to alanine (C to A), and transfected these DNA constructs into HEK293 cells followed by SNP treatment. As demonstrated in Fig. 3D, SNP increased S-nitrosylated levels of His-AMPKγ1-WT, His-AMPKγ1-C38A, and His-AMPKγ1-C279A, but not His-AMPKγ1-C131A. Accordingly, AMPK catalytic activity in HEK293 cells expressing His-AMPKγ1-WT, His-AMPKγ1-C38A or His-AMPKγ1-C279A were inhibited by SNP. While, His-AMPKγ1-C131A was resistant to SNP (Fig. 3E).

### Homocysteine thiolactone (HTL) or high glucose (HG) decreases AMPK activity in VSMCs through AMPKγ1 S-nitrosylation

Next, we determined whether metabolic risk factors HTL and HG, also as cardiovascular risk factors (Chen et al, 2021; Li et al, 2016; Yu et al, 2014), decreased AMPK activity through NO-mediated AMPKγ1 S-nitrosylation. We generated lentivirus harboring mutant of mouse AMPKγ1 by replacing C130 to alanine (MT-AMPKγ1-C130A), which is equal to human 131 (a gain-of-function mutant) and called S-nitrosylation-resistant AMPKγ1, and infected lentivirus into murine VSMCs. As shown in Appendix Fig. S4a,b, compared to cells without infection or infected with empty vector, both exogenous His-AMPKγ1-WT and His-AMPKγ1-MT dramatically increased total AMPKγ1 expressions. Compared to cells without infection, virus infection lightly affected cell viability, while both AMPKγ1-WT and AMPKγ1-MT did not further affect cell viability in basal condition (Appendix Fig. S4c). Either HTL or HG induced AMPKγ1 S-nitrosylation in VSMCs infected with lentivirus expressing WT-AMPKγ1 but not MT-AMPKγ1 (Appendix Fig. S5a,b). Conversely, AMPK activity was decreased after HTL or HG incubation in VSMCs expressing WT-AMPKγ1, while AMPK activity was not reduced by HTL or HG if VSMCs expressed S-nitrosylation-resistant AMPKγ1 (Appendix Fig. S5c).

To examine if the desensitization of AMPK to AMP contributes to HTL or HG-reduced AMPK activity in VSMCs, we pretreated human VSMCs with AICAR, an adenosine analog taken up into cells to generate AMP-mimetic actions (Kim et al, 2016). As illustrated in Appendix Fig. S5d, AICAR alone markedly increased AMPK activity in basal conditions. However, AICAR had no effects on AMPK activity if cells were treated with HTL or HG. Additionally, canonical AMPK stimuli, such as AICAR, phenformin and glucose starvation, did not lead to of AMPK S-nitrosylation (Appendix Fig. S5e).

### HTL or HG via nitrosative stress inactivates AMPK in VSMCs

**Figure 4:**
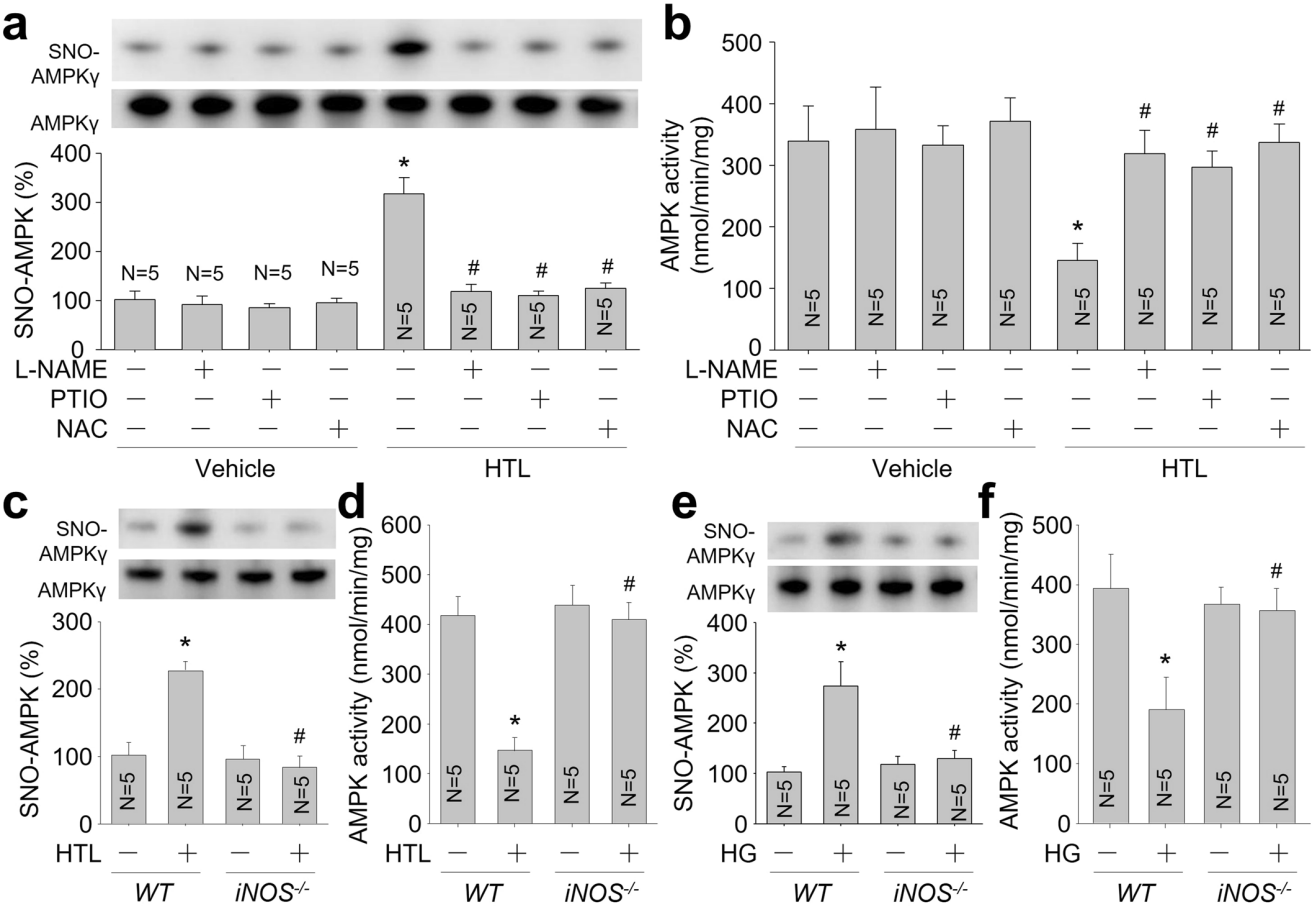
Homocysteine thiolactone (HTL) or high glucose (HG) via the iNOS/NO signaling induces AMPKγ S-nitrosylation in VSMCs. (**A**) Cultured human VSMCs were pretreated with N’-nitro-L-arginine-methyl ester (L-NAME, 1 mM), or carboxyl-PTIO (PTIO, 0.3 mM), or N-acetyl-cysteine (NAC, 2.5 mM) for 30 minutes followed by incubation with HTL (1 mM) for 24 h. Purified AMPKγ1 protein from cell lysates was subjected to measure AMPKγ1 S-nitrosylation. (**B**) AMPKα protein in cell lysates was purified by using anti-AMPKα antibody. (**C**) Primary VSMCs isolated from *WT* and *iNOS*^*-/-*^ mice were incubated with HTL (1 mM) for 24 h. Total cell lysates were subjected to measure AMPKγ1 S-nitrosylation. (**D**) Total cell lysates were subjected to measure AMPK activity. (**E**) *WT* and *iNOS*^*-/-*^ VSMCs were incubated with HG (30 mM) for 24 h. Total cell lysates were subjected to measure AMPKγ1 S-nitrosylation. (**F**) Total cell lysates were subjected to measure AMPK activity. Error bars are mean ± SEM. *P < 0.05 vs. Vehicle alone. ^#^*P* < 0.05 vs. HTL alone in (**A**, **B**). *P < 0.05 vs. Vehicle-treated *WT* cells. ^#^*P* < 0.05 vs. HTL-treated *WT* cells in (**C**, **D**). *P < 0.05 vs. Vehicle-treated *WT* cells. ^#^*P* < 0.05 vs. HG-treated *WT* cells in (**E**, **F**). A one-way ANOVA followed by Tukey post hoc tests was used to determine *P* value.

Under HHcy, endogenous NO mainly derived from inducible NO synthase (iNOS) (Zhang et al, 2017b). To determine whether HTL via iNOS-mediated nitrosative stress inactivates AMPK, cells were treated with iNOS inhibitor N’-nitro-L-arginine-methyl ester (L-NAME), NO cleaner carboxyl-PTIO, and NAC. Though HTL induced AMPKγ1 S-nitrosylation and reduced AMPK activity in human VSMCs, PTIO, L-NAME, and NAC abolished AMPKγ1 S-nitrosylation and reversed AMPK activity in HTL-treated cells (Fig. 4A,B).

To exclude any potential off-target effects of L-NAME, we determined if genetic deletion of iNOS mimicked the effects of L-NAME on AMPKγ1 S-nitrosylation in VSMCs. Primary VSMCs isolated from *WT* mice and *iNOS*^*-/-*^ mice were incubated with HTL or HG. They increased AMPKγ1 S-nitrosylation and decreased AMPK activity in *WT* cells, but not in *iNOS*^*-/-*^ cells (Fig. 4C–F).

### HTL or HG promotes VSMC phenotype switching to senescence via iNOS and AMPKγ1

**Figure 5:**
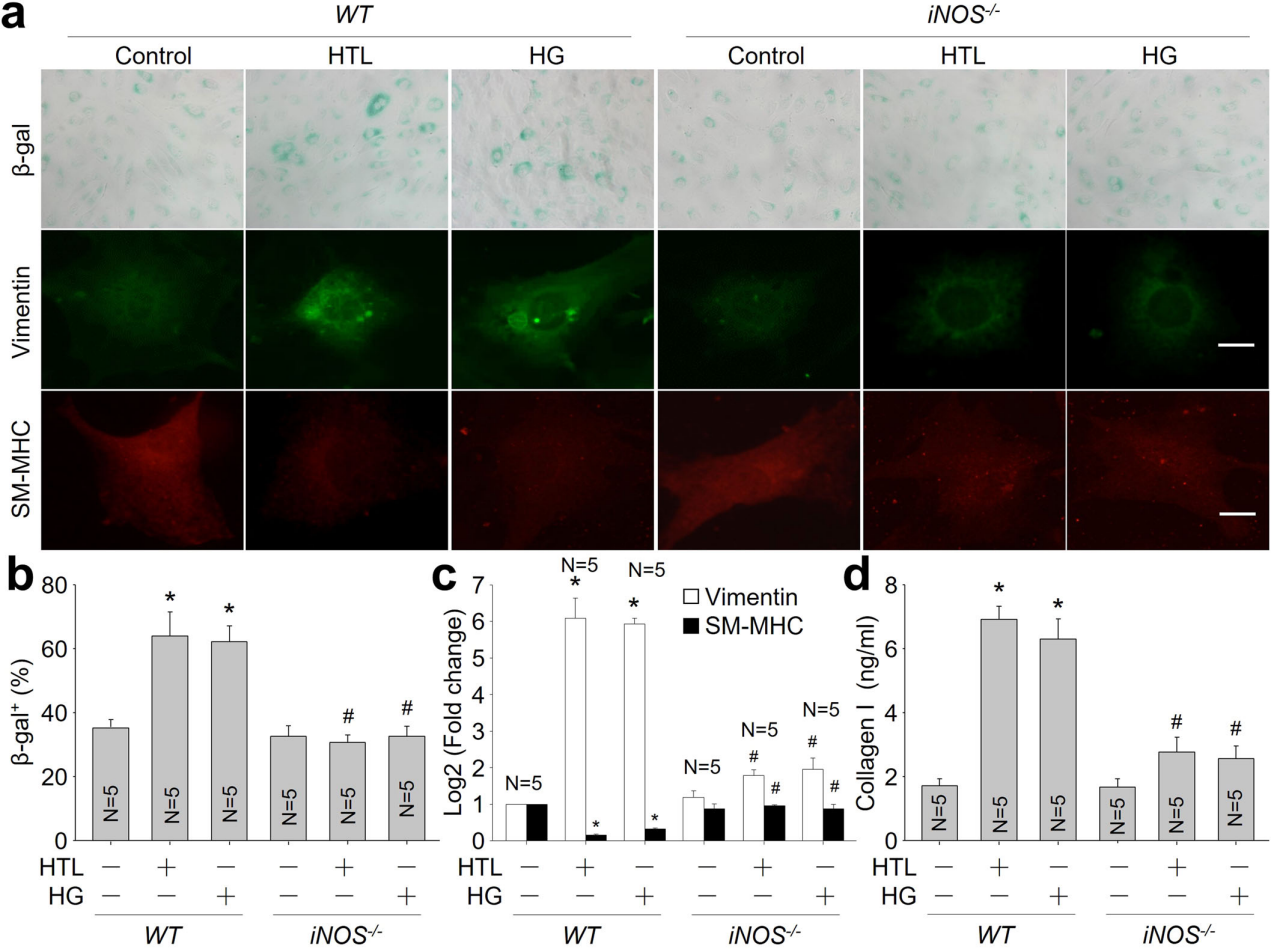
Homocysteine thiolactone (HTL) or high glucose (HG) promotes VSMC senescence through iNOS. (**A**) Primary VSMCs isolated from *WT* and *iNOS*^*-/-*^ mice were incubated with HTL (1 mM) or HG (30 mM) for 24 h. Cells were subjected to perform β-galactosidase staining and IFC analysis of vimentin or SM-MHC. (**B**) Quantitative analysis of β-galactosidase activity was performed. (**C**) Gene expressions of vimentin and SM-MHC were conducted using quantitative PCR. (**D**) The level of collagen I in culture medium was determined by ELISA. The scale bar represents 10 µm. Error bars are mean ± SEM. *P < 0.05 vs. WT alone. ^#^*P* < 0.05 vs. WT plus HTL or HG. A one-way ANOVA followed by Tukey post hoc tests was used in (**B**–**D**).

**Figure 6:**
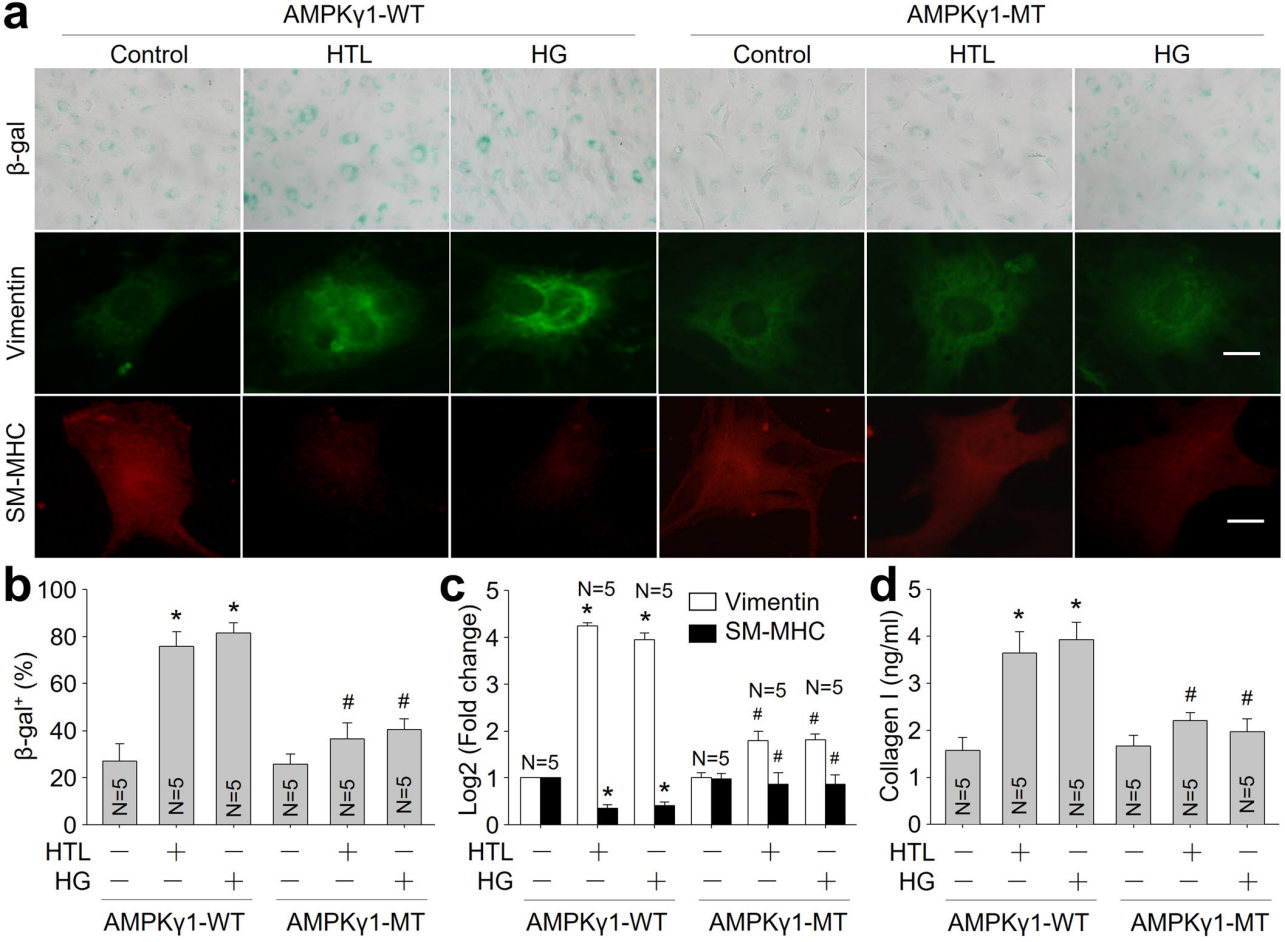
Homocysteine thiolactone (HTL) or high glucose (HG) induces VSMC phenotypic switching through AMPKγ S-nitrosylation. (**A**) Murine VSMCs were infected with lentivirus expressing WT-AMPKγ1 or MT-AMPKγ1 (C130A) for 48 h followed by incubation with HTL (1 mM) or HG (30 mM) for 24 h. Cells were subjected to perform β-galactosidase staining and IFC analysis of vimentin or SM-MHC. (**B**) Quantitative analysis of β-galactosidase staining was performed. (**C**) Gene expressions of vimentin and SM-MHC were conducted using quantitative PCR. (**D**) The level of collagen I in culture medium was determined by ELISA. The scale bar represents 10 µm. Error bars are mean ± SEM. *P < 0.05 vs. AMPKγ1-WT alone. ^#^*P* < 0.05 vs. AMPKγ1-WT plus HTL or HG. A one-way ANOVA followed by Tukey post hoc tests was used in (**B**–**D**).

We next investigated if HTL or HG promotes VSMC senescence through iNOS-mediated nitrosative stress. As presented, either HTL or enhanced NO production, β-galactosidase positive staining, collagen I secretion and vimentin expression, but decreased the expressions of SM-MHC, p27 and p21 in VSMCs isolated from *WT* mice, rather than VSMCs isolated from *iNOS*^*-/-*^ mice (Fig. 5A–D and Appendix Fig. S6a,b). These effects produced by iNOS gene deletion were mimicked by exogenous expression of MT-AMPKγ1 in murine VSMCs (Fig. 6A–D and Appendix Fig. S6c,d).

### VSMC-specific iNOS knockout alleviates ischemia-induced cardiac injury in *Apoe*^*-/-*^ mice with HHcy or hyperglycemia

Knowing the importance of iNOS-mediated nitrosative stress in VSMC phenotype switching in vitro, we had to determine the role of VSMC-specific iNOS in the maturation of coronary collateral artery in vivo. To this end, we generated VSMC-specific *Apoe*^*-/-*^*/iNOS*^*sm-/-*^ mice and established the RI/MI model (Appendix Fig. S7a). HHcy was mimicked by feeding mice with HTL and hyperglycemia was induced by STZ. The plasma levels of lipids, glucose, and homocysteine were presented in Appendix Table S2. In *Apoe*^*-/-*^ mice following RI/MI, both HHcy and hyperglycemia dramatically increased the infarction size of hearts (Appendix Fig. S7b,c) and plasma cTn-I level (Appendix Fig. S7d), and induced nitrosative stress (Appendix Fig. S8a,b), and worsened cardiac dysfunctions (Appendix Table S2). While, these detrimental effects of HHcy and hyperglycemia were disappeared in *Apoe*^*-/-*^*/iNOS*^*sm-/-*^ mice.

### HHcy and hyperglycemia impair coronary collateral circulation via VSMC-specific iNOS in vivo

**Figure 7:**
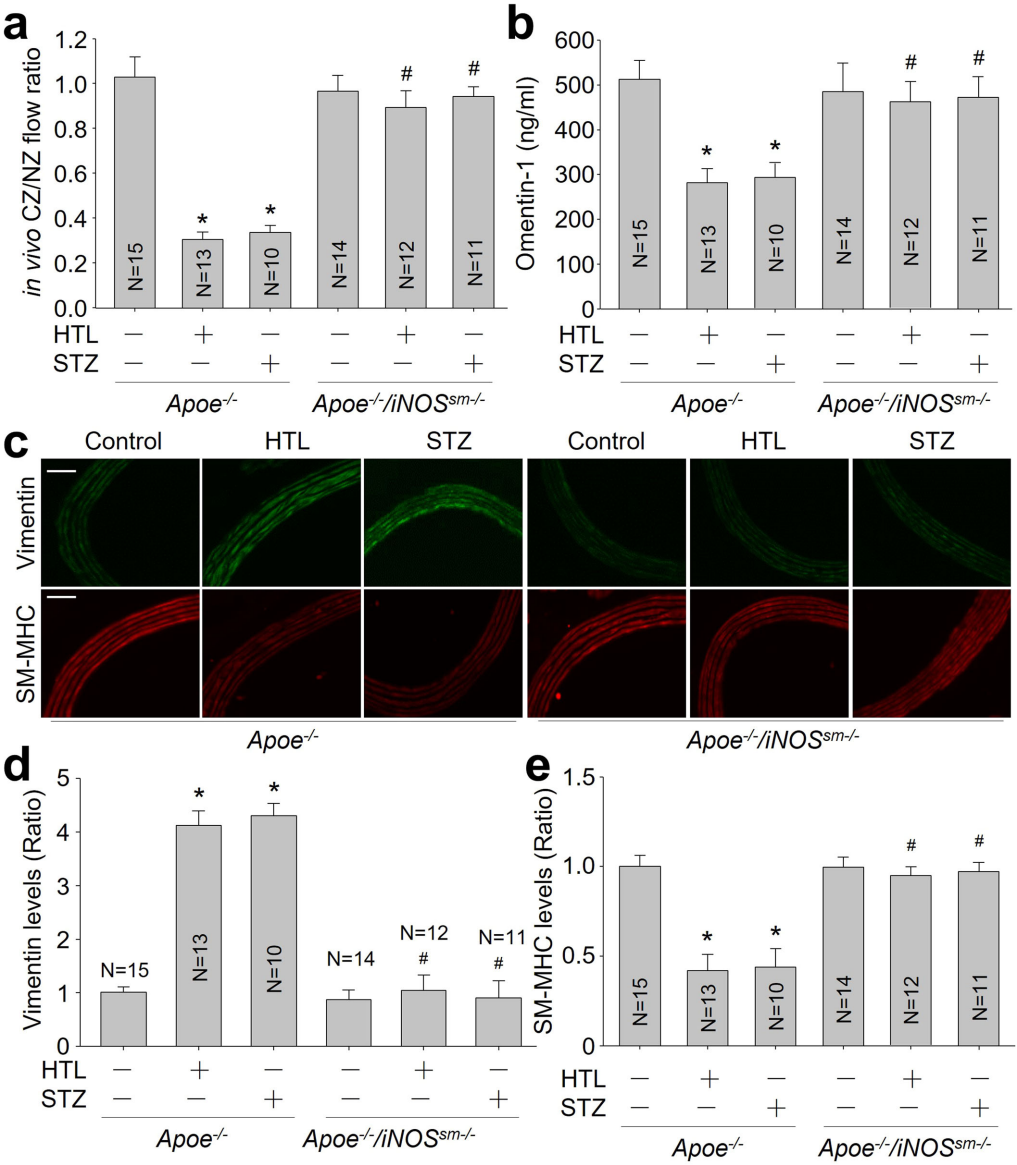
VSMC-specific iNOS knockout restores vascular contractile phenotype and improves coronary collateral circulation in *Apoe*^*-/-*^ mice with hyperhomocysteinemia or hyperglycemia. (**A**) The protocols and experimental designs were described in Appendix Fig. S7a. Coronary blood flow was measured in collateral zone (CZ) and normal zone (NZ) using microspheres, and in vivo coronary collateral blood flow (CCBF) was expressed as the ratio of CZ/NZ flow. (**B**) Plasma omentin-1 level was assayed using ELISA. (**C**) The coronary artery was subjected to perform IFC analysis of contractile marker SM-MHC or synthetic marker vimentin. (**D**) Quantitative analyses of vimentin. (**E**) Quantitative analyses of SM-MHC. The scale bar represents 20 µm. Error bars are mean ± SEM. *P < 0.05 vs. Apoe^*-/-*^ mice. ^#^*P* < 0.05 vs. Apoe^*-/-*^ mice plus HTL or STZ. A one-way ANOVA followed by Tukey post hoc tests was used in (**A**, **B**, **D**, **E**).

As expected, HHcy and hyperglycemia significantly decreased CCBF (Fig. 7A) and ex *vivo* coronary flow (Appendix Table S2), lowered serum omentin-1 levels (Fig. 7B), and inhibited the maturation of pre-existing arteries in collateral area and vascular contractile phenotypic restoration (Fig. 7C–E) in *Apoe*^*-/-*^ mice following MI, rather than *Apoe*^*-/-*^*/iNOS*^*sm-/-*^ mice. Biotin-switched method analysis indicated that S-nitrosylated levels of AMPKγ1 protein were remarkably increased in *Apoe*^*-/-*^ mice with HHcy and hyperglycemia, as well as AMPK activity reduction if compared to control *Apoe*^*-/-*^ mice. These alterations induced by HHcy and hyperglycemia were not observed in *Apoe*^*-/-*^*/iNOS*^*sm-/-*^ mice (Appendix Fig. S8c,d).

### S-nitrosylation-resistant AMPKγ1 improves coronary collateral circulation in *Apoe*^*-/-*^ mice with HHcy or hyperglycemia

**Figure 8:**
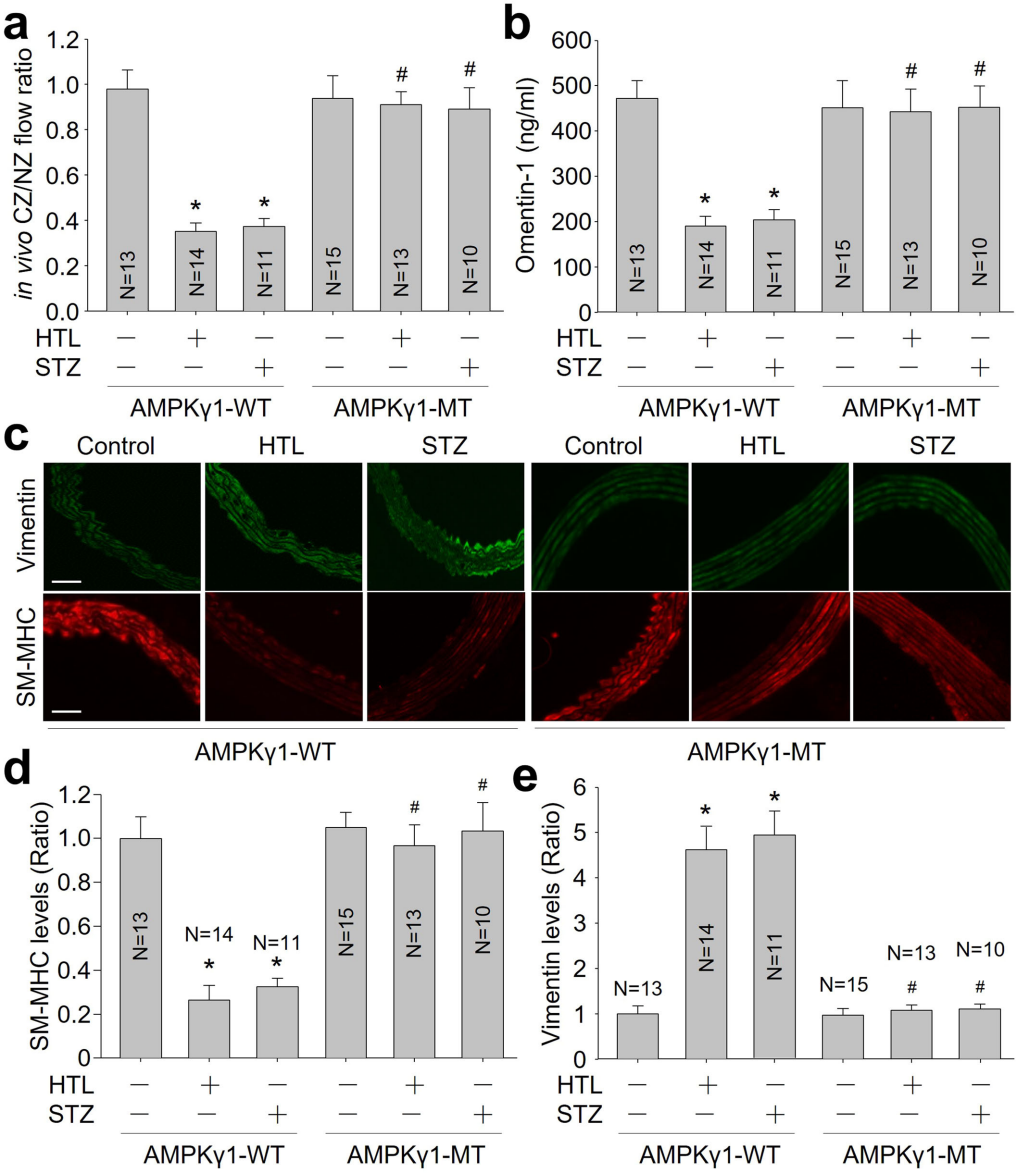
Exogenous expression of S-nitrosylation-resistant AMPKγ1 enhances vascular contractile phenotypic restoration and coronary collateral circulation in *Apoe*^*-/-*^ mice with hyperhomocysteinemia or hyperglycemia. (**A**) Coronary blood flow was measured in collateral zone (CZ) and normal zone (NZ) using microspheres, and in vivo coronary collateral blood flow (CCBF) was expressed as the ratio of CZ/NZ flow. (**B**) Plasma omentin-1 level was assayed using ELISA. (**C**) The coronary artery was subjected to perform IFC analysis of contractile marker SM-MHC or synthetic marker vimentin. (**D**) Quantitative analyses of SM-MHC. (**E**) Quantitative analyses of vimentin. The scale bar represents 20 µm. Error bars are mean ± SEM. *P < 0.05 vs. WT-AMPKγ1. ^#^*P* < 0.05 vs. WT-AMPKγ1 plus HTL or STZ. A one-way ANOVA followed by Tukey post hoc tests was used in (**A**, **B**, **D**, **E**).

To clarify the in vivo role of AMPKγ1 S-nitrosylation in the maturation of coronary collateral artery, we infected *Apoe*^*-/-*^ mice with adeno-associated virus 9 expressing WT-AMPKγ1 or MT-AMPKγ1 (Appendix Fig. S9a). As depicted in Appendix Fig. S9b, AAV9-mediated exogenous expression of WT-AMPKγ1 or MT-AMPKγ1 was clearly observed in VSMCs in the heart. HHcy or hyperglycemia increased infraction size and plasma cTn-I level (Appendix Fig. S10a–c), and promoted heart dysfunctions (Appendix Table S3) in *Apoe*^*-/-*^ mice expressing WT-AMPKγ1. Further, they decreased *ex vivo* coronary flow (Appendix Table S3) and CCBF (Fig. 8A), reduced serum omentin-1 level (Fig. 8B), and prevented vascular contractile phenotype restoration (Fig. 8C–E) in *Apoe*^*-/-*^ mice expressing WT-AMPKγ1, but not in *Apoe*^*-/-*^ mice expressing MT-AMPKγ1 mice. As expected, HHcy and hyperglycemia increased AMPKγ1 S-nitrosylation and decreased AMPK activity in *Apoe*^*-/-*^ mice expressing WT-AMPKγ1, rather than MT-AMPKγ1 (Appendix Fig. S10d,e). Besides, neither the *Apoe*^*-/-*^ mouse nor AAV9 are specific to VSCMs and some phenotypes may in part be caused by changes to macrophages or cardiomyocytes, respectively.

### Poor coronary collateral circulation and increases AMPKγ1 S-nitrosylation in patients with HHcy or diabetes

To provide translational perspectives of this study, we conducted a pilot experiment to determine the association between coronary collateral circulation and nitrosative stress in patients with MI. The demographic data of human subjects were presented in Appendix Table S4. As presented, both Rentrop grades and plasma omentin-1 levels in patients with HHcy or diabetes were lower than patients without HHcy or diabetes (Appendix Fig. S11a,b), while plasma cTn-I levels were higher (Appendix Fig. S11c).

We finally assayed AMPKγ1 S-nitrosylation and AMPK activity in leukocytes isolated from these patients. As shown in Appendix Fig. S11d,e, AMPK activity was decreased in patients with HHcy or diabetes, while AMPKγ1 S-nitrosylation was upregulated. Importantly, there was a positive correction between AMPK activity and Rentrop grade (*r* = 0.8979, Appendix Fig. S11f). Although the pilot experiment did not establish the cause-effect link between nitrosative stress and coronary collateral circulation, it still implies the important role of AMPKγ1 S-nitrosylation in the maturation of coronary collateral artery in patients with HHcy or diabetes after acute MI.

## Discussion

The major finding of our studies is that nitrosative stress is a novel mechanism of poor coronary collateral circulation in patients following acute MI. Current treatments of acute MI have focused on increasing NO bioavailability to induce coronary arterial relaxation (Munzel and Gori, 2013; Tian et al, 2018). However, continuous exposure to nitrate esters can lead to nitrate tolerance, causing the loss of vascular responsiveness to nitrates (Bai et al, 2018; Zhou et al, 2019). Our findings provide a mechanistic explanation for the failure of NO approaches as therapeutic strategies in patient with HHcy or diabetes through impairing coronary collateral circulation. This conclusion is supported by several observations. First, nitrosative stress by NTG continuous infusion decreased CCBF and aggravated cardiac dysfunctions in rats following MI, which were abolished by inhibition of protein S-nitrosylation. Second, HHcy and hyperglycemia, liking NTG, reduced CCBF and promoted ischemia-induced cardiac dysfunctions in *Apoe*^*-/-*^ mice but not in *Apoe*^*-/-*^*/iNOS*^*sm-/-*^ mice. Third, VSMC reprogramming, a key step for building coronary collateral circulation after acute MI, was delayed by NTG, HHcy or hyperglycemia in vitro and in vivo. Therefore, we suggest that nitrate therapy should be reconsidered in populations with HHcy or hyperglycemia. In those patients, iNOS suppression and nitrosative stress alleviation may be a promising treatment for acute MI because the iNOS-mediated nitrosative stress contributes to poor coronary collateral circulation.

Another important finding is that AMPK activity is regulated by NO-directed AMPKγ S-nitrosylation. Protein post-translational modifications, including phosphorylation, ubiquitination, glycation, etc., are important to determine protein functions and play key roles in many cellular processes (Bah and Forman-Kay, 2016). In general, AMPK catalytic activity is majorly controlled by phosphorylation at threonine 172 by upstream kinase such as LKB1 and CaMKK, as reported previously (Steinberg and Kemp, 2009). This study provides new evidence to support the proposal that AMPK function is greatly related to AMPKγ1 S-nitrosylation under nitrosative stress. This discovery uncovers a novel mechanism of AMPK post-translational modification.

It has been known that AMPK deficiency contributes to the initiation and progression of atherosclerosis-associated diseases (Salt and Hardie, 2017). In ischemic heart, AMPK activation is essential for post-ischemic angiogenesis, leading to new capillaries and cardiac repairs (Daskalopoulos et al, 2016). In this study, we demonstrated that AMPK performs anti-ischemic action by building collateral circulation, consistent with an earlier study that endothelial AMPK activation in endothelial cells improves coronary flow responses in vivo (Enkhjargal et al, 2014). However, we further extended this observation by showing that VSMC-specific AMPK activation reprograms VSMCs to promote the maturation of collateral artery to provide blood flow resupply through coronary collateral circulation. Based on our observations, AMPK might be an attractive target for therapeutically intervening arterial occlusive diseases.

It is well recognized that collaterals develop through two distinct stages including growth and maturation. During collateral growth, the phenotype of vascular smooth muscle cell (VSMC) is proliferative. In the stage of collateral maturation, VSMC phenotype is characteristically contractile. Based on our observations, a transition exists between growth and maturation, in which VSMC phenotype returns from proliferative to contractile. Thus, we conceptually reasoned that collaterals development undergoes three stages including growth, conversion, and maturation. Moreover, VSMC reprogramming during transition is critical to determine the function of collateral circulation. Under hyperhomocysteinemia and hyperglycemia, nitrosative stress impairs VSMC reprogramming to restrict the formation of functional collateral artery. This viewpoint may explain why patients with metabolic syndrome have poor coronary collateral circulation after acute MI.

Usually, the strategy of coronary blood flow rebuilding after MI is the invasive reperfusion therapy, such as percutaneous coronary intervention and coronary artery bypass grafting, as the first choice of most cardiologists. However, this extremely depends on the time to hospital arrival and the basic conditions of patients (Guan et al, 2019; Moser et al, 2006). Moreover, the presence of restenosis is inevitable. This finding is medically relevant because we suggest a new approach to save life by taking drugs to inhibit AMPK S-nitrosylation immediately once acute chest pain occurs in patients who are not hospitalized, even it is a considerable medicine for patients who are not suitable for invasive operations. In long-term, VSMC reprogramming by nitrosative stress inhibition or AMPK activation, as a new non-invasive approach, might be an adjuvant therapy or a substitute of the surgery-based reperfusion therapy, due to its convenience, efficacy, and compliance.

In conclusion, HHcy and hyperglycemia trigger the iNOS-mediated nitrosative stress to prevent VSMC phenotypic restoration through AMPKγ1 S-nitrosylation. In this way, metabolic risk factors deteriorate coronary collateral circulation to aggravate ischemia-induce cardiac injury (Appendix Fig. S12).

## Methods

### Materials

Nitroglycerin (NTG), sodium nitroprusside (SNP), streptozotocin (STZ), homocysteine thiolactone (HTL), 5-aminoimidazole-4-carboxamide1-β-D-ribofuranoside (AICAR), N’-nitro-L-arginine-methyl ester hydrochloride (L-NAME), N-acetyl-cysteine (NAC), and carboxyl-PTIO (PTIO) were purchased from Sigma-Aldrich Company (Merck KGaA, Darmstadt, Germany). Primary antibodies against inducible NO synthase (iNOS), AMPKα, vimentin, smooth muscle myosin heavy chain (SM-MHC), and GAPDH were purchased from Cell Signaling Transduction Company. Protein A/G plus-agarose and secondary antibody were obtained from Santa Cruz Biotechnology Inc. (Santa Cruz, CA). Cellular senescence assay kit was bought from Cell Biolabs (San Diego, CA, USA). Recombinant human AMPKγ1 protein (ab132975), recombinant human AMPKα1β1γ1 protein (ab79803), anti-AMPKγ1 antibody (ab223116), and biotin switch assay kit (S-Nitrosylation) (ab236207) were obtained from Abcam Company (San Francisco, USA). AMPK substrate SAMS peptide (Amino acid sequence: HMRSAMSGLHLVKRR, Cat. 1344) was purchased from Bio-Techne China Co. Ltd (Shanghai, China). Commercial kits for determinations of collagen I ELISA Kit (ab210579), cardiac troponin I (cTn-I) ELISA Kit (ab200016), and omentin-1 ELISA Kit (ab269545) were purchased from Abcam Company (San Francisco, USA). Lentivirus (LV) or adeno-associated virus 9 (AAV9) of AMPKγ cDNA were generated by Shanghai Genechem Co., Ltd. (Shanghai, China). All drug concentrations are expressed as final working concentrations in the buffer.

### Animals and generations of nitrosative stress, hyperhomocysteinemia (HHcy), and diabetes

Male Sprague-Dawley rats (8 weeks old, 180–200 g) were provided by the Experimental Animal Center of Henan Province. Male wildtype (*WT*) C57B16, *iNOS*^*-/-*^, *Apoe*^*-/-*^, and VSMC-specific iNOS knockout *Apoe*^*-/-*^ (*Apoe*^*-/-*^/*iNOS*^*sm-/-*^) mice at 10–12 weeks of age with 20–25 g body weight were used in this study. *WT* mice, *iNOS*^*-/-*^ mice, and SM22α-Cre knock-in homozygous (*SM22α*^*Cre/Cre*^) mice were obtained from Jackson Laboratories (Bar Harbor, ME). *iNOS*^*flox/flox*^ mice were generated by us. *iNOS*^*sm-/-*^ mice were generated by crossing *iNOS*^*flox/flox*^ mice with *SM22α*^*Cre/Cre*^ transgenic mice. *iNOS*^*sm-/-*^ mice were crossed with *Apoe*^*-/-*^ mice to generate *Apoe*^*-/-*^*/iNOS*^*sm-/-*^ mice. This study was carried out in accordance with the ethical standards laid down in the 1964 Declaration of Helsinki and its later amendments. The animal protocol was reviewed and approved by the Animal Care and Use Committee, Qilu Hospital, University of Shandong University.

For the establishment of nitrosative stress model, rats were continuously infused with NTG (50 mg/kg/day, 5 days) by using Alzet osmotic pumps (DURECT Corp.) as describe previously (Wang et al, 2012). For the induction of hyperglycemia (Liu et al, 2015), a low-dose STZ (50 mg/kg/day, 5 consecutive days, I.P.) was used to induce pancreatic islet cell destruction and persistent hyperglycemia as described by the Animal Models of Diabetic Complications Consortium (http://www.amdcc.org). For the establishment of HHcy, mice were intragastrically gavaged with HTL (50 mg/kg/day) for 4 weeks as described previously (Yang et al, 2015).

### RI model

Rats or mice were used for chronic (0–12 days) implantation of a pneumatic occluder over the left anterior descending coronary artery (LAD) as described previously (Toyota et al, 2005). A suture was passed under the proximal portion of the LAD and the occluder was sown onto the surface of the heart. The occlude catheter was externalized between the scapulae. When the occlude is inflated, the suture is pulled towards the surface of the heart and the LAD is occluded. The LAD perfusion territory is termed the collateral-dependent zone (CZ) because perfusion in this area, while the LAD is occluded, depends on the development of coronary collaterals (Appendix Fig. S13).

### MI model

We let the occlude be inflated permanently based on the RI model or ligated LAD without thoracotomy as we described previously (Sun et al, 2018).

### In vivo measurements of CCBF using microsphere

As described previously (Yin et al, 2012), microspheres (5 × 10^5^) were injected into the left ventricle lumen via a 30-G needle over 20 s during LAD occlusion at day 0 (the initial of RI) and at day 12 (the conclusion of each experiment). Samples dissected from the collateral-dependent zone (CZ) and normal zone (NZ) are weighed and activity during neutron activation of the spheres was measured (BioPAL, Worcester, MA). Collateral flow was calculated as a ratio between activity (dpm/g) of the tissues from the CZ and NZ (CZ/NZ).

### TTC staining and quantification of infarct size

As described previously (DeGeorge et al, 2008), the heart was rapidly excised 24 h after surgical procedures and then washed for three times in cold PBS. The detailed method used to determine the infarct size was shown in Appendix Fig. S14.

### IFC and IHC

As described previously (Wu et al, 2018; Yu et al, 2016), s sections were deparaffinized, rehydrated, and blocked with 5% normal serum. The ratio used in the analysis of IFC image means the relative intensity of fluorescence. The absolute intensities of each group measured by Alpha Ease FC software were normalized by the absolute intensities of control group.

### Protein S-nitrosylation assay

As described previously (Zhou et al, 2019), proteins were extracted according to the manufacturer’s specification S-Nitrosylated Protein Detection Assay Kit (Cayman, USA), which is based on the “Biotin-switch” method.

### Generations of DNA constructs

WT-AMPKγ1 cDNA were purchased from Origene Company. Cysteine residues were replaced with alanine by using the QuikChange kit (Stratagene), according to the manufacturer’s instructions.

### Virus infections to cells or animals

Cells were infected with lentivirus overnight in antibiotics-free medium supplemented with 2% FBS. For infecting mice, AAV9 containing WT-AMPKγ1 or MT-AMPKγ1 cDNA was injected via tail vein under pressure in 1 ml of PBS with 7.6 × 10^7^ IFUs of loaded virus. The concentration of DNA was 10 mg/kg.

### Cell cultures

Human VSMCs purchased from ATCC or primary murine VSMC isolated from *WT* mice and *iNOS*^*-/-*^ mice were grown in Smooth Muscle Cell Medium (Sciencell, USA) supplemented with 2% fetal bovine serum, penicillin (100 U/ml) and streptomycin (10 mg/ml).

### Senescence-associated β-galactosidase staining

As described previously (Yin et al, 2022), the cells were fixed with 4% paraformaldehyde for 15 min and was stained with acidic β-galactosidase.

### Western blotting

As described previously (Chen et al, 2021), tissues were homogenized on ice in cell-lysis buffer. Total 20 µg proteins were loaded to SDS-PAGE. The background was subtracted from the calculated area.

### AMPK activity assay

AMPK activity was assayed by using the SAMS peptide as previously described (Witters and Kemp, 1992; Zhang et al, 2017a).

### Relative RNA quantifications by RT-qPCR

As described previously (Li et al, 2016), total RNA was isolated using a TRIzol-based (Invitrogen)RNA isolation protocol. The primer sequences were shown in Appendix Fig. S15. Relative gene expression is determined using the comparative threshold cycle (*C*_T_) method as described previously (Schmittgen and Livak, 2008). The fold change for mRNA expression was calculated using the formula 2^ΔΔCT^. The basal level of target gene expression in control group was set as 1 (2^0^).

### Detection of intracellular NO

NO production in culture cells was detected using the fluorescent probe DAF as described previously (Li et al, 2019a).

### Measurements of blood glucose, cholesterol, triglyceride, cTn-I, omentin-1, homocysteine and HTL

The blood levels of glucose, cholesterol, triglyceride, cTn-I, and omentin-1 in blood were assayed using commercial kits as recommend by the providers. The determination of HTL has been described previously (Jakubowski, 2002). Plasma levels of homocysteine were measured by highly selective analytical methods like HPLC combined with fluorescence detection as described previously (Smith et al, 2012).

### Patients and sample processing

Coronary angiography was routinely performed for all patients using standard Judkins technique to determine the grade of coronary collateral circulation according to the Rentrop classification (Rentrop et al, 1985). Informed consent was obtained from all participants. The procedures were in accordance with the ethical standards of the responsible committee on human experimentation or with the Helsinki Declaration of 1975.

### Statistical analysis

The data among multiple groups were analyzed with a one-way ANOVA followed by Tukey post hoc tests. The data obtained from the time/concentration courses were analyzed with repeated-measures ANOVA following by multiple comparisons between two groups. Statistical analyses were conducted using GraphPad Prism 6.0. A two-sided *P* value < 0.05 was considered as significance.

### Supplementary information

Appendix

## Data Availability

This study includes no data deposited in external repositories. Data are available upon request.
