# Supplementary material for: S-nitrosylation of AMPKγ impairs coronary collateral circulation and disrupts VSMC reprogramming
Source: EMBO Rep. 2023 Dec 18;25(1):128–43. doi: 10.1038/s44319-023-00015-3 (PMC10897329; doi:10.1038/s44319-023-00015-3)
Supplement: Supplementary file 1 — Appendix [file 44319_2023_15_MOESM1_ESM.pdf]

Contents

**Items**	**Page Number**
Appendix Figure S1	2
Appendix Figure S2	4
Appendix Figure S3	6
Appendix Figure S4	7
Appendix Figure S5	8
Appendix Figure S6	10
Appendix Figure S7	11
Appendix Figure S8	13
Appendix Figure S9	14
Appendix Figure S10	15
Appendix Figure S11	17
Appendix Figure S12	18
Appendix Figure S13	19
Appendix Figure S14	20
Appendix Figure S15	21
Appendix Table S1	22
Appendix Table S2	23
Appendix Table S3	24
Appendix Table S4	25

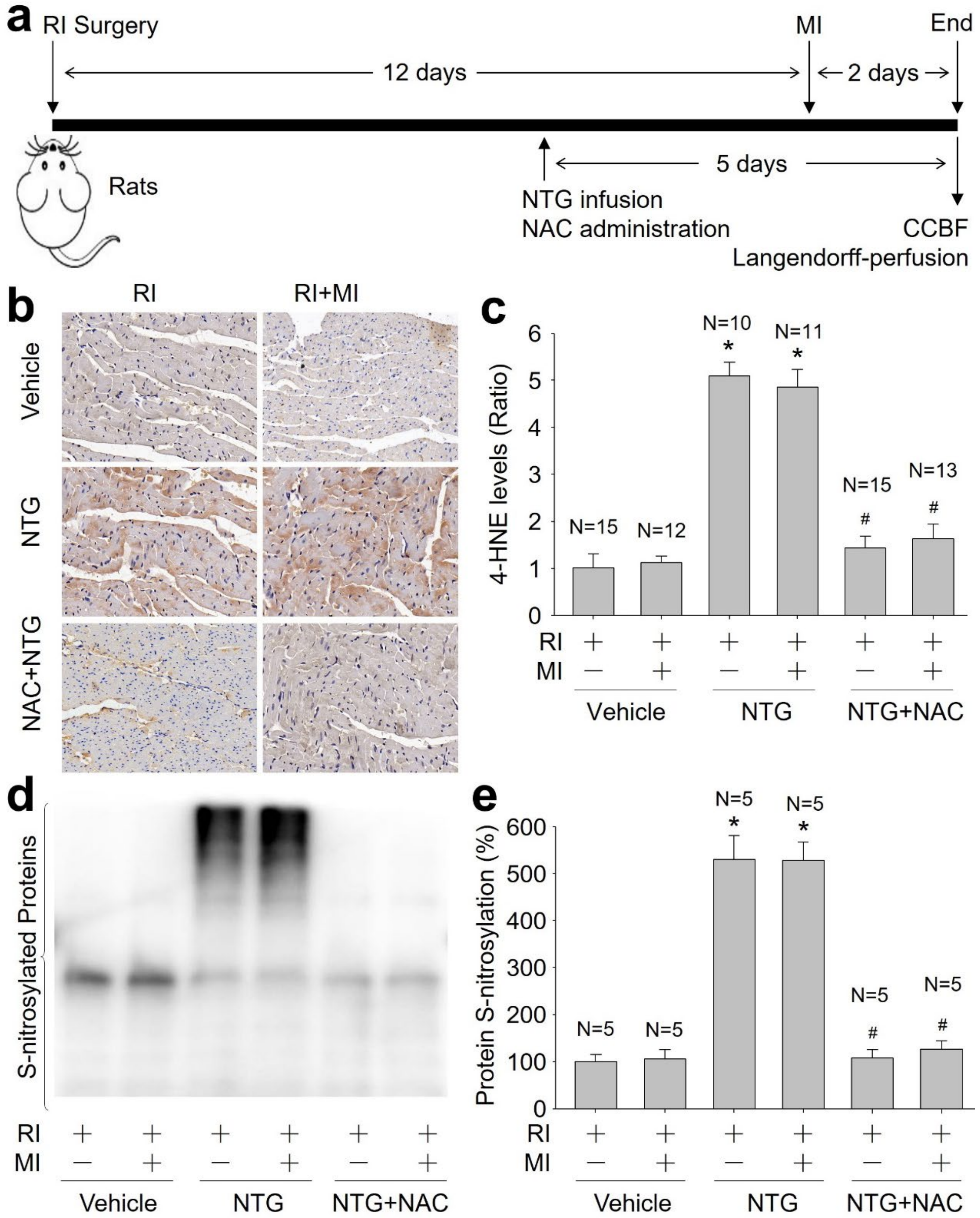

Appendix Figure S1. Continuous infusion of nitroglycerin (NTG) induces nitrosative stress in rats with RI/MI, which are abolished by N-acetyl-cysteine (NAC). (**a**) Rats were subjected to perform RI surgery for 12 days to induce the growth of coronary collateral artery followed by MI surgery. One week after RI surgery, rats were continuously infused with in NTG (50 mg/kg/day) for 5 days using Alzet osmotic pumps. Two days after MI surgery, coronary collateral blood flow (CCBF) was measured using microsphere before sacrificed. Hearts isolated from rats were subjected to measure

coronary flow and heart functions using Langendorff-perfused hearts. (**b** and **c**) IHC analysis of 4-Hydroxynonenal (4-HNE) was performed in heart tissue isolated from collateral zone in **b** and quantitative analysis was performed in **c**. (**d** and **e**) Homogenates of heart collateral zones were subjected to measure protein S-nitrosylation using biotin-switched method in **d** and the quantitative analysis was performed in **e**. $^{*}P < 0.05$ *vs.* vehicle plus RI or RI/MI. $^{\#}P < 0.05$ *vs.* NTG plus RI or RI/MI. A one-way ANOVA followed by Tukey *post-hoc* tests was used to determine *P* value in **c** and **e**.

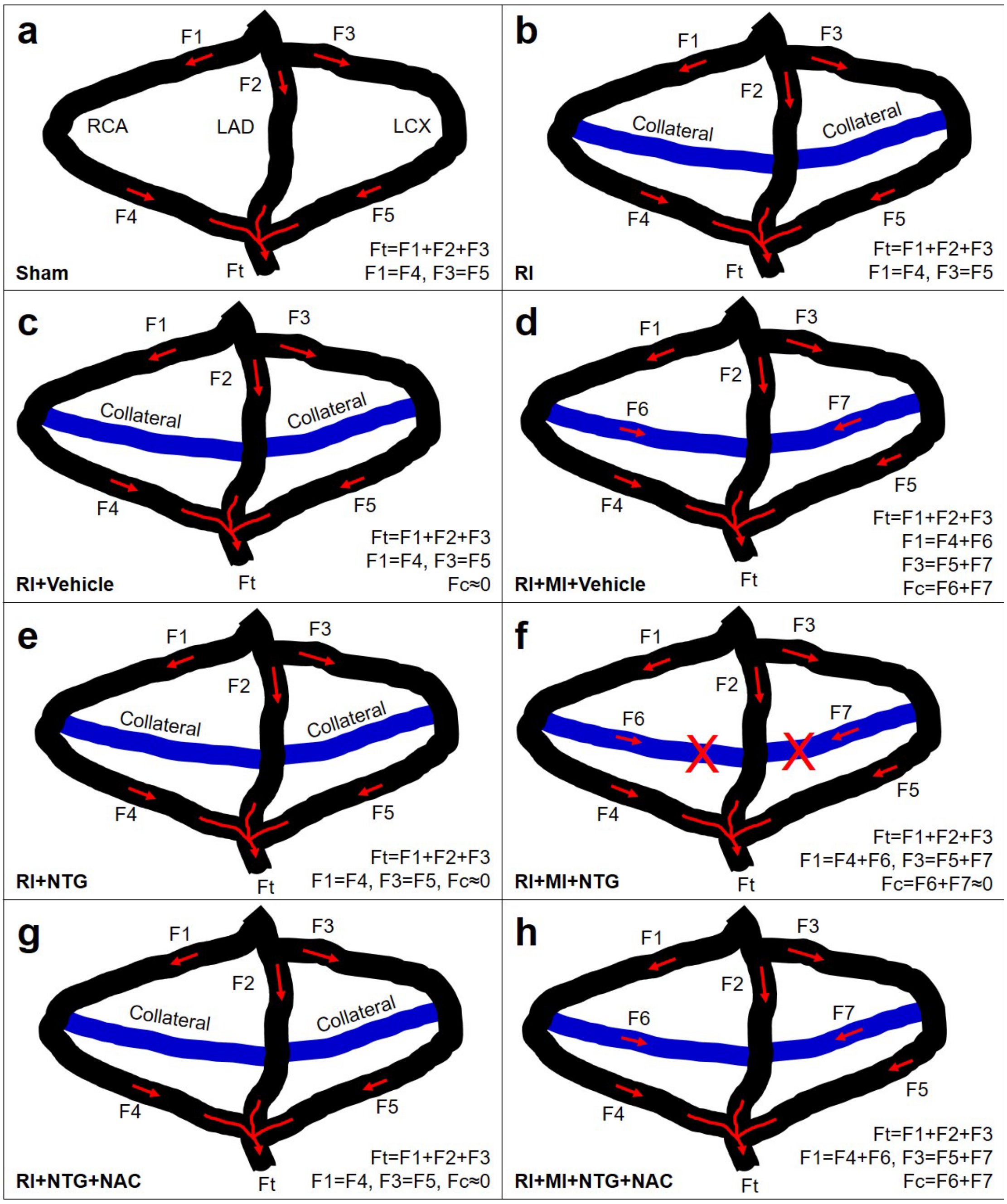

Appendix Figure S2. Illustrations of coronary blood flow in rats with or without collateral circulation. The protocols and experimental designs were described in Appendix Fig S1a. (**a**) There is no collateral artery growth in rats without RI surgery. Under this condition, total coronary blood flow (Ft) is the sum of blood flows in RCA (F1), LAD (F2), and LCX (F3). (**b** and **c**) RI surgery induces the growth of coronary collateral artery in rats treated with vehicle or not. In this case, coronary collateral artery is closed without blood flow. Coronary collateral blood flow (Fc) is close to zero. (**d**) Collateral artery has grown in rats with RI surgery. MI triggers the maturation of collateral artery

due to pressure gradient. (**e**) Rats with RI surgery have grown coronary collateral artery, while these coronary collateral arteries do not open because these is no occlusion. (**f**) NTG inhibits the maturation of collateral artery. (**g**) Coronary blood flows in these rats are similar with rats in **c** and **e**. (**h**) NAC combats with the effects of NTG to promote the maturation of collateral artery. Ft is reversed, compared to rats in **f**. LAD, left anterior descending. RCA, right coronary artery. LCX, left circumflex branch. Red X indicates blocked. Red arrow represents blood flow. Blue line means collateral artery.

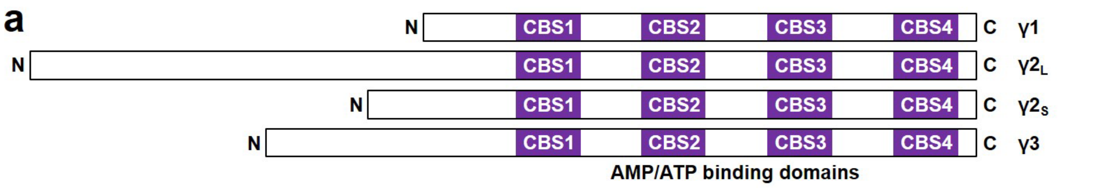

b
1 metvissdss pavenehpqe tpesnnsvyt sfmkshr**c**yd liptssklvv fdtslqvkka
61 ffalvtngvr aaplwdskkq sfvgmltitd finilhryyk salvqiyele ehkietwrev
121 ylqdsfkplv **c**ispnaslfd avsslirnki hrlpvidpes gntlyilthk rilkflklfi
181 tefpkpefms ksleelqigt yaniamvrtt tpvyvalgif vqhrvsalpv vdekgrvvdi
241 yskfdvinla aektynnldv svtkalqhrs hyfegvlk**c**y lhetletiin rlveaevhrl
301 vvvdendvvk givslsdilq alvltggekk p

c
1 mesvaaessp alenehfqet pesnnsvyts fmkshr**c**ydl iptssklvvf dtslqvkkaf
61 falvtngvra aplwdskkqs fvgmltitdf inilhryyks alvqiyelee hkietwrevy
121 lqdsfkplv**c** ispnaslfda vsslirnkih rlpvidpesg ntlyilthkr ilkflklfit
181 efpkpefmsk slqelqigty aniamvrttt pvyvalgifv qhrvsalpvv dekgrvvdiy
241 skfdvinlaa ektynnldvs vtkalqhrsh yfegvlk**c**yl hetletiinr lveaevhrlv
301 vvdehdvvkg ivslsdilqa lvltggekkp

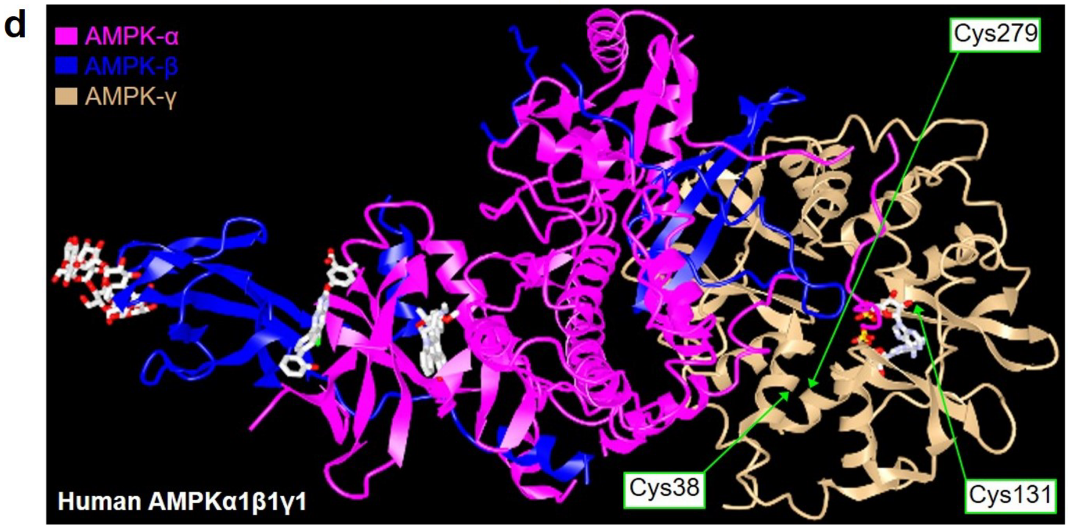

Appendix Figure S3. The amino acid sequence and structure of AMPKγ1 protein. (**a**) Schematic representation of AMPKγ components. Three isoforms of γ subunit have variable N-terminal domains. Four cystathione-β-synthases (CBS) domains form two Bateman domains that create four adenosine nucleotide-binding sites. (**b**) Human AMPKγ1 protein contains 3 cysteine residues, which locate in the 38th, 131th, and 279th of amino acids. (**c**) Murine AMPKγ1 protein contains 3 cysteine residues, which locate in the 37th, 130th, and 278th of amino acids. (**d**) The γ subunit is for the regulation within the AMPK complex as it is solely responsible for the enzyme's ability to acutely sense AMP/ATP levels within the cell. Three cysteines (38, 131, 279) are illustrated. Cysteine 38 and 279 are close, while cysteine 131 is very close to the AMP binding site.

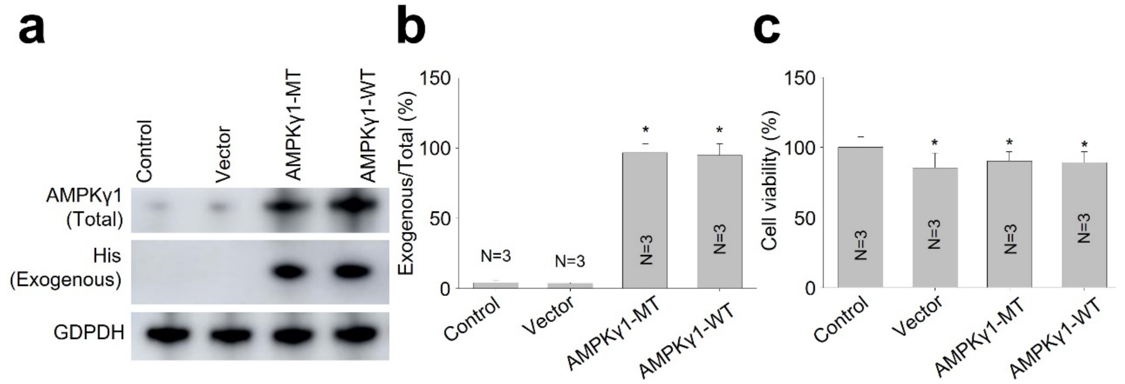

Appendix Figure S4. Effects of exogenous AMPKγ1 in cell viability of VSMCs. (**a**) VSMCs were infected with virus expressing His-tagged WT-AMPKγ1 or MT-AMPKγ1 (C130A) for 48 hours. Total cell lysates were subjected to perform western blot analysis of total AMPKγ1 and His expressions. (**b**) Exogenous AMPKγ1 protein was calculated in **a**. Exogenous AMPKγ1 was defined as the ratio of His to total AMPKγ1. (**c**) Cells were subjected to assay cell viability by MTT. $^{*}P<0.05$ *vs.* control or vector. A one-way ANOVA followed by Tukey *post-hoc* tests was used to determine *P* value in **b** and **c**.

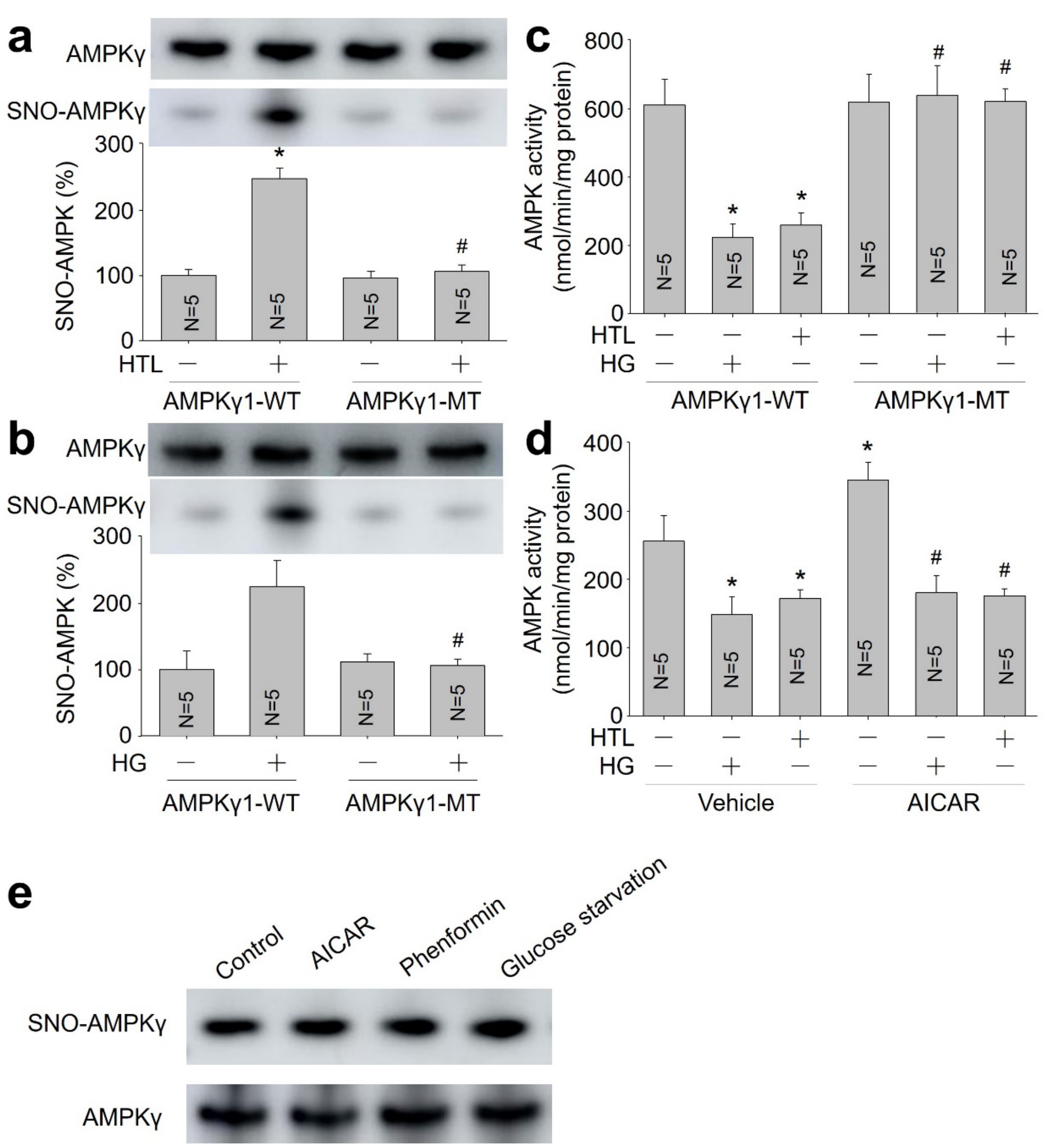

Appendix Figure S5. Homocysteine thiolactone (HTL) or high glucose (HG) induce AMPKγ1 S-nitrosylation to inhibit AMPK activity in VSMCs. (**a-c**) Primary murine VSMCs were infected with lentivirus expressing His-AMPKγ1-WT or His-AMPKγ1-MT (C130A, equal to C131A in human) for 48 hours followed by incubation with HTL (1 mM) in **a** or high glucose (HG, 30 mM) in **b** for 24 hours. His-AMPKγ1 protein in cell lysates purified from total cell lysates was subjected to measure AMPKγ1 S-nitrosylation. AMPK activity was assayed in **c**. **P*<0.05 *vs.* AMPKγ1-WT alone. $^{\#}$*P*<0.05 *vs.* AMPKγ1-WT plus HTL or HG. (**d**) Cultured human VSMCs were pretreated with AICAR (1 mM) for 30 minutes followed by incubation with HTL (1 mM) or HG (30 mM) for 24 hours. Total cell lysates were subjected to measure AMPK activity. (**e**) Cultured human VSMCs were pretreated with AICAR (1 mM), phenformin (2

mM), and glucose starvation for 24 hours. Total cell lysates were subjected to measure AMPKγ1 S-nitrosylation using biotin-switch method. $^{*}P<0.05$ *vs.* Vehicle alone. $^{\#}P<0.05$ *vs.* AICAR alone. A one-way ANOVA followed by Tukey *post-hoc* tests was used to determine *P* value in **c** and **d**.

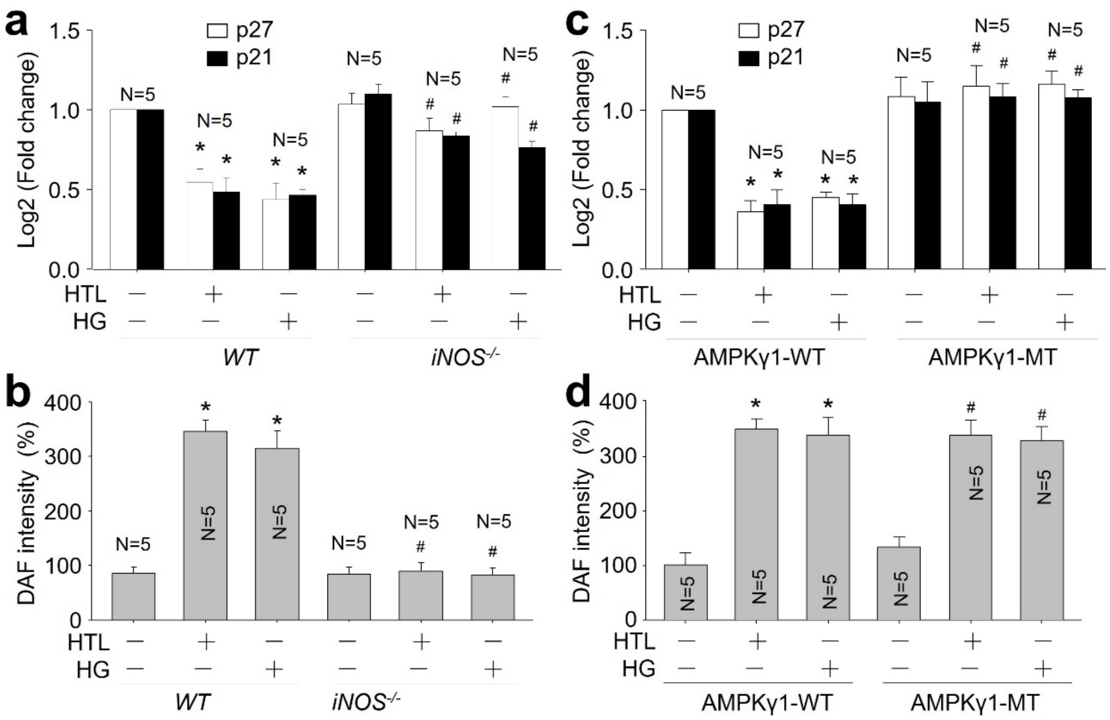

Appendix Figure S6. Homocysteine thiolactone (HTL) or high glucose (HG) increases iNOS-derived NO production in VSMCs. (**a** and **b**) Primary VSMCs isolated from *WT* mice and *iNOS*$^{-/-}$ mice were incubated with HTL (1 mM) or HG (30 mM) for 24 hours. Gene expressions of p21 and p27 were conducted using quantitative PCR in **a**. Intracellular NO was assayed using DAF fluorescence intensity in **b**. $^{*}P<0.05$ *vs. WT* alone. $^{\#}P<0.05$ *vs. WT* plus HTL or HG. (**c** and **d**) Murine VSMCs were infected with lentivirus expressing WT-AMPKγ1 or MT-AMPKγ1 (C130A) for 48 hours followed by incubation with HTL (1 mM) or HG (30 mM) for 24 hours. Gene expressions of p21 and p27 were conducted using quantitative PCR in **c**. Intracellular NO was assayed using DAF fluorescence intensity in **d**. $^{*}P<0.05$ *vs.* AMPKγ1-WT alone. $^{\#}P<0.05$ *vs.* AMPKγ1-MT alone. A one-way ANOVA followed by Tukey *post-hoc* tests was used to determine *P* value in this figure.

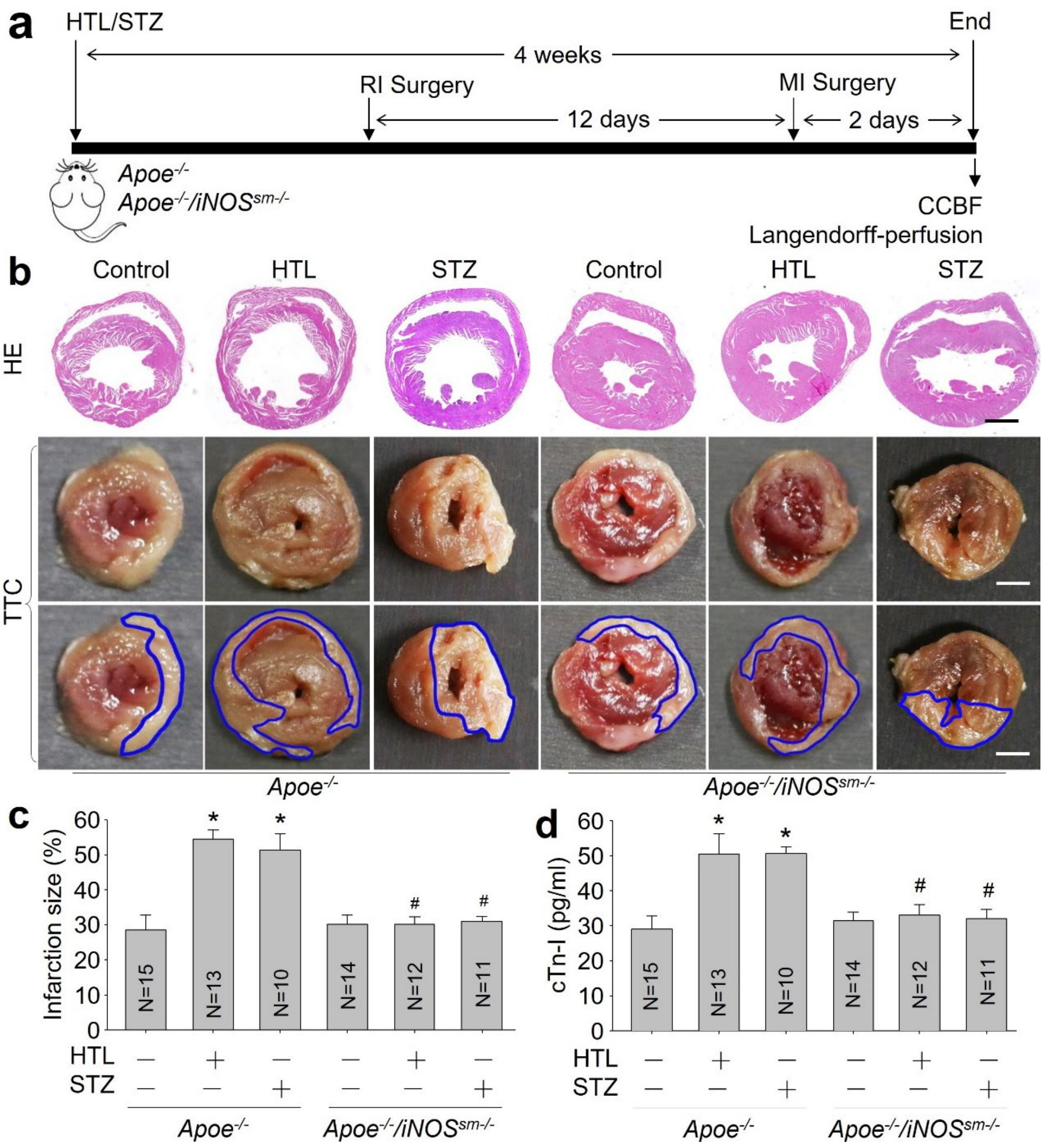

Appendix Figure S7. VSMC-specific iNOS knockout diminished the infarction size in *Apoe*$^{-/-}$ mice with hyperhomocysteinemia or hyperglycemia. (**a**) Hyperhomocysteinemia were mimicked by feeding *Apoe*$^{-/-}$ mice and *Apoe*$^{-/-}$/*iNOS*$^{sm-/-}$ mice with homocysteine thiolactone (HTL). Hyperglycemia was induced by injecting mice with streptozotocin (STZ). RI surgery was performed for 12 days to induce the coronary collateral artery growth followed by MI surgery for 2 days. Coronary collateral blood flow (CCBF) was measured using microsphere before sacrificed. Hearts isolated from rats were subjected to measure coronary flow and heart functions using Langendorff-perfused hearts. (**b**) The morphology of heart was determined by HE or TTC staining. The infarction area was circled by blue line. (**c**)

Quantitative analysis of infarction area was performed in pictures shown in **b**. (**d**) Plasma cTn-I level was determined in mice. The scale bar represents 500 μm. $^{*}P < 0.05$ *vs. Apoe*$^{-/-}$ mice. $^{\#}P < 0.05$ *vs. Apoe*$^{-/-}$ mice plus HTL or STZ. A one-way ANOVA followed by Tukey *post-hoc* tests was used to determine *P* value in **c** and **d**.

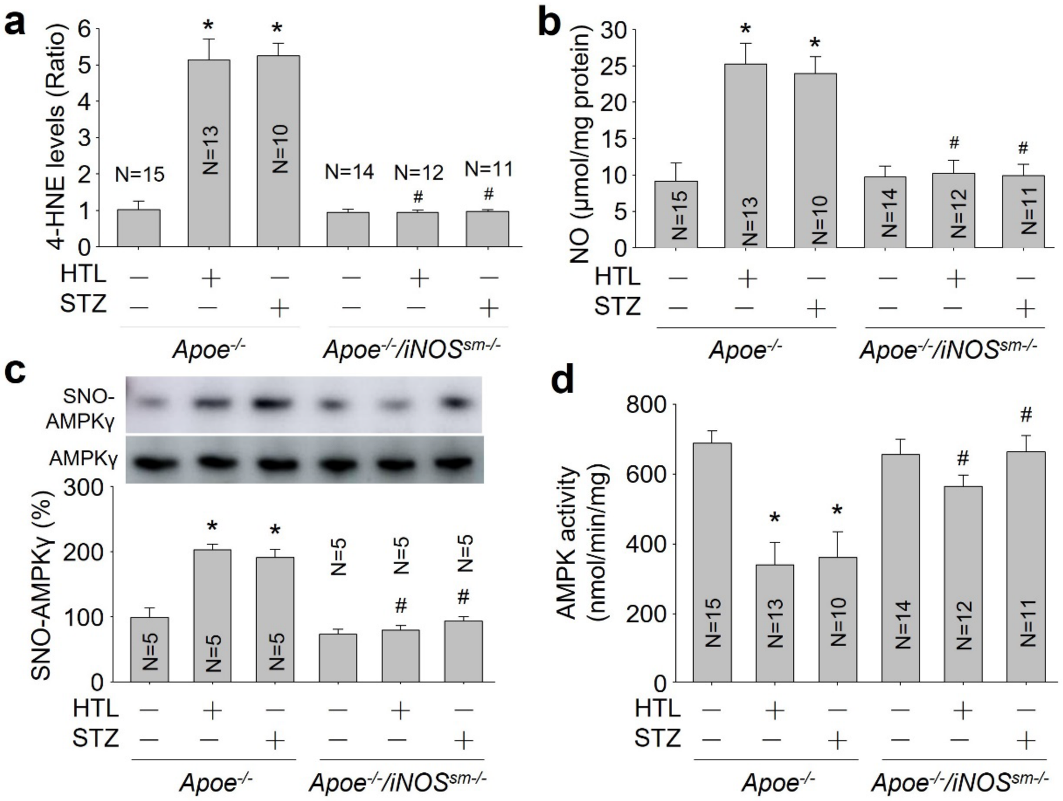

Appendix Figure S8. VSMC-specific iNOS knockout abolishes AMPKγ1 S-nitrosylation in *Apoe*$^{-/-}$ mice with hyperhomocysteinemia or hyperglycemia. The protocols and experimental designs were described in Appendix Fig S7a. (**a**) IHC and quantitative analysis of 4-HNE in pictures shown in **a**. (**b**) NO content in heart tissue isolated from collateral zone was measured using Gries method. (**c** and **d**) AMPKγ1 S-nitrosylation in **c** and AMPK activity in **d** were assayed in heart tissues isolated from collateral zone. $^{*}P < 0.05$ *vs. Apoe*$^{-/-}$ mice. $^{\#}P < 0.05$ *vs. Apoe*$^{-/-}$ mice plus HTL or STZ. A one-way ANOVA followed by Tukey *post-hoc* tests was used to determine *P* value in this figure.

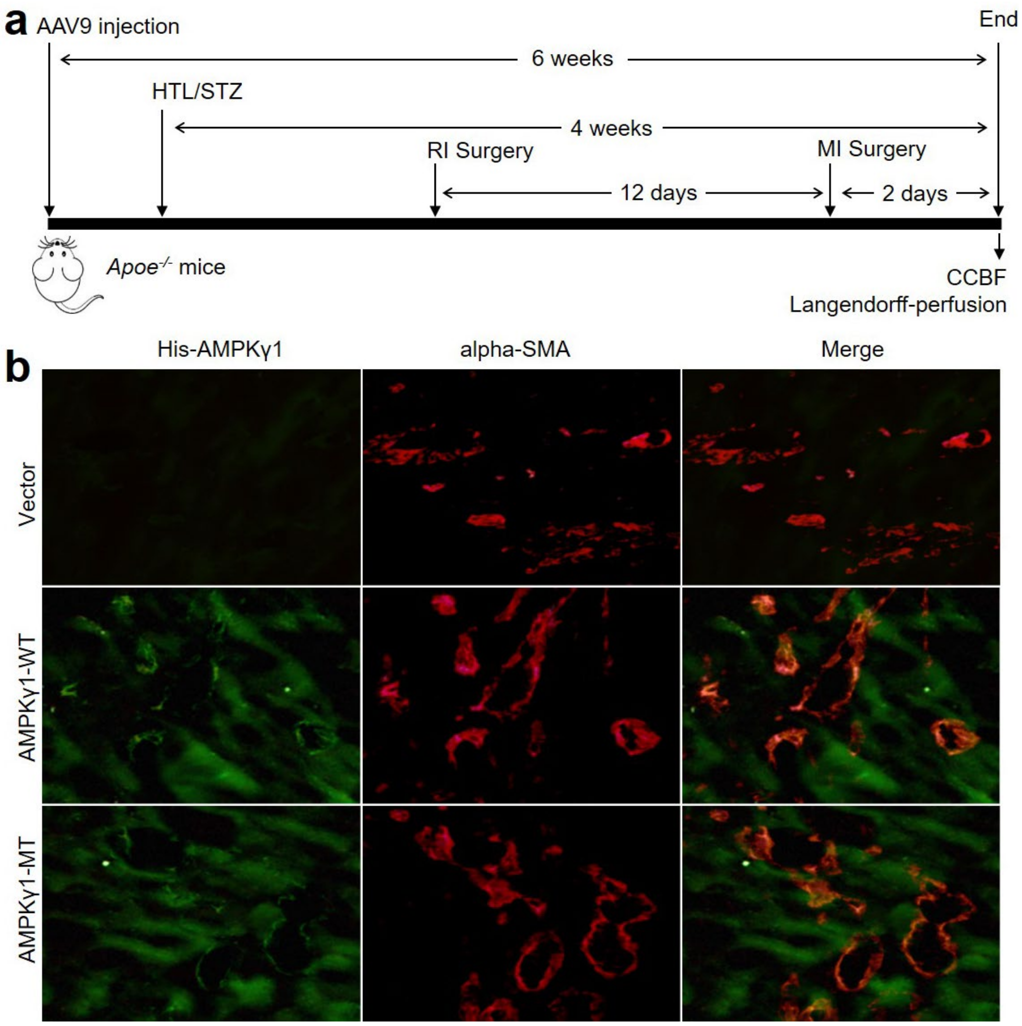

Appendix Figure S9. Exogenous expression of S-nitrosylation-resistant AMPKγ1 in *Apoe*$^{-/-}$ mice with hyperhomocysteinemia or hyperglycemia following RI/MI. (**a**) *Apoe*$^{-/-}$ mice were infected with AAV9 expressing WT-AMPKγ1 or MT-AMPKγ1-C130A via tail vein injection once per 4 weeks. Hyperhomocysteinemia was mimicked by feeding mice with homocysteine thiolactone (HTL). Hyperglycemia was induced by injecting mice with streptozotocin (STZ). RI surgery was performed for 12 days to induce the coronary collateral artery growth followed by MI surgery for 2 days. Coronary collateral blood flow (CCBF) was measured using microsphere before sacrificed. Hearts isolated from rats were subjected to measure coronary flow and heart functions using Langendorff-perfused hearts. (**b**) The area of collateral zone isolated from heart was subjected to perform IFC analysis of His-AMPKγ1 or VSMC marker α-SMA.

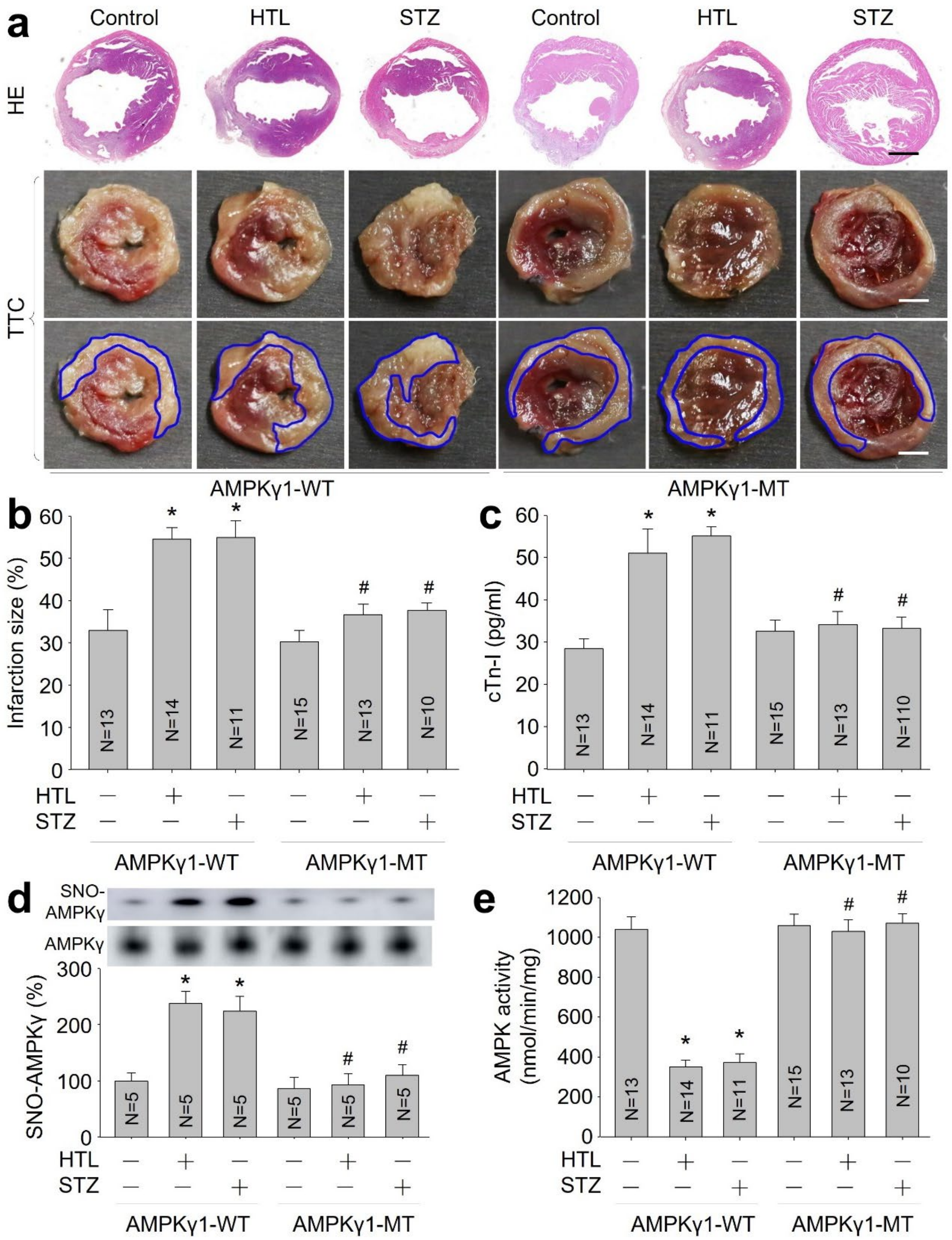

Appendix Figure S10. Exogenous expression of S-nitrosylation-resistant AMPKγ1 reduced the infarction size and preserves AMPK activity in *Apoe*$^{-/-}$ mice with hyperhomocysteinemia or hyperglycemia following RI/MI. The protocols and experimental designs were described in Appendix Fig S9a. (**a**) The morphology of heart was determined by HE or TTC staining. The infarction area was circled by blue line. (**b**) Quantitative analysis of infarction area was performed in pictures shown in **a**. (**c**) Plasma cTn-I level

was determined in mice. (**d** and **e**) AMPKγ1 S-nitrosylation in **d** and AMPK activity in **e** were assayed in heart tissues isolated from collateral zone. The scale bar represents 500 μm. **P* < 0.05 *vs.* WT-AMPKγ1. $^{\#}$*P* < 0.05 *vs.* WT-AMPKγ1 plus HTL or STZ. A one-way ANOVA followed by Tukey *post-hoc* tests was used to determine *P* value in **b-e**.

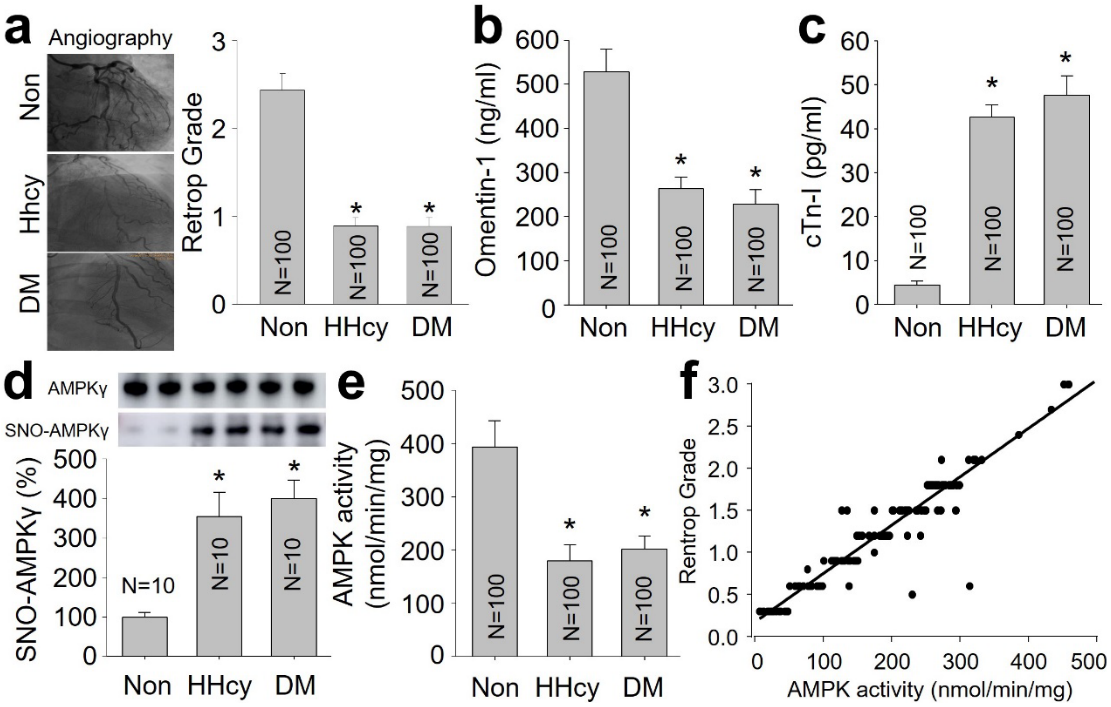

Appendix Figure S11. Poor collateral circulation, increased AMPKγ S-nitrosylation, and reduced AMPK activity in patients with hyperhomocysteinemia (HHcy) or diabetes (DM). The demographic data of human subjects were presented in Appendix Tab S4. (**a**) Coronary collateral circulation was visualized by angiography in human patients with acute MI. Quantitative analysis of collateral circulation was performed using Rentrop Grade. (**b** and **c**) Plasma levels of omentin-1 in **b** and cTn-1 in **c** were assayed using ELISA. (**d** and **e**) Total cell lysates of leucocytes from peripheral blood were subjected to determine AMPKγ1 S-nitrosylation by biotin-switched method in **d** and ATP^{32}-SMAS peptide method in **e**. (**f**) Correction analysis between AMPK activity and Rentrop Grade was performed. $^{*}P < 0.05$ *vs.* patients without HHcy or DM. A one-way ANOVA followed by Tukey *post-hoc* tests was used to determine *P* value in **a-e**.

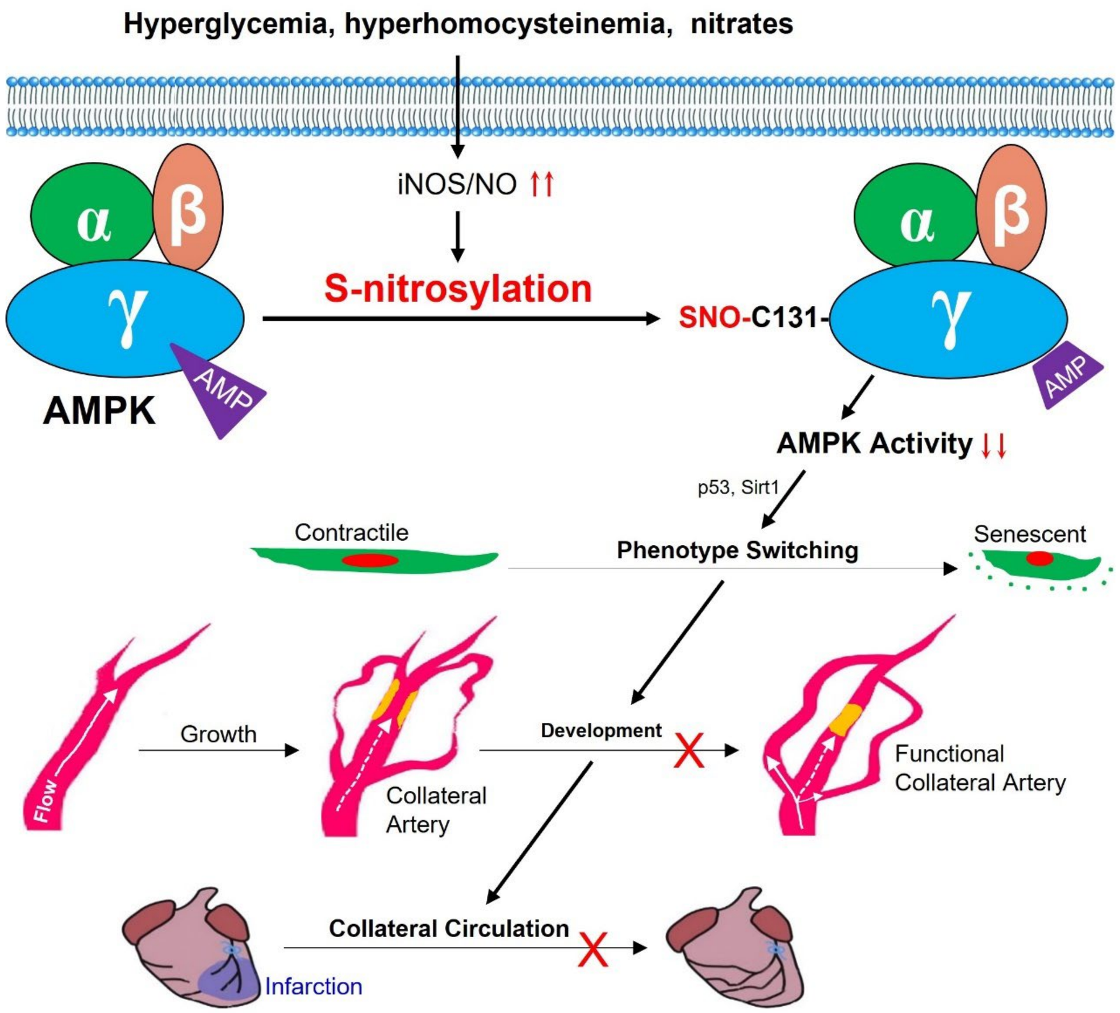

Appendix Figure S12. Roles of nitrosative stress in coronary collateral circulation. Transient, repetitive ischemia triggers VSMC proliferation and phenotypic switching from the contractile to the synthetic, which stimulates coronary collateral growth without blood flow (immature arteriogenesis). Once occlusion occurs, VSMCs immediately return to the contractile phenotype to let collateral artery open (mature arteriogenesis) due to gradient pressure. Under nitrosative stress, hyperhomocystinemia and hyperglycemia induce iNOS positively expressed in vascular smooth muscles of the pre-existing collateral arteries to produce excessive NO. Then, NO directly S-nitrosylates AMPKγ protein to desensitize AMP, resulting in AMPK suppression. Deficient AMPK promotes VSMC senescence, which prevents phenotype restoration from synthetic to contractile. In this way, nitrosative stress inhibits the maturation of the pre-existing collateral artery to destroy coronary collateral circulation.

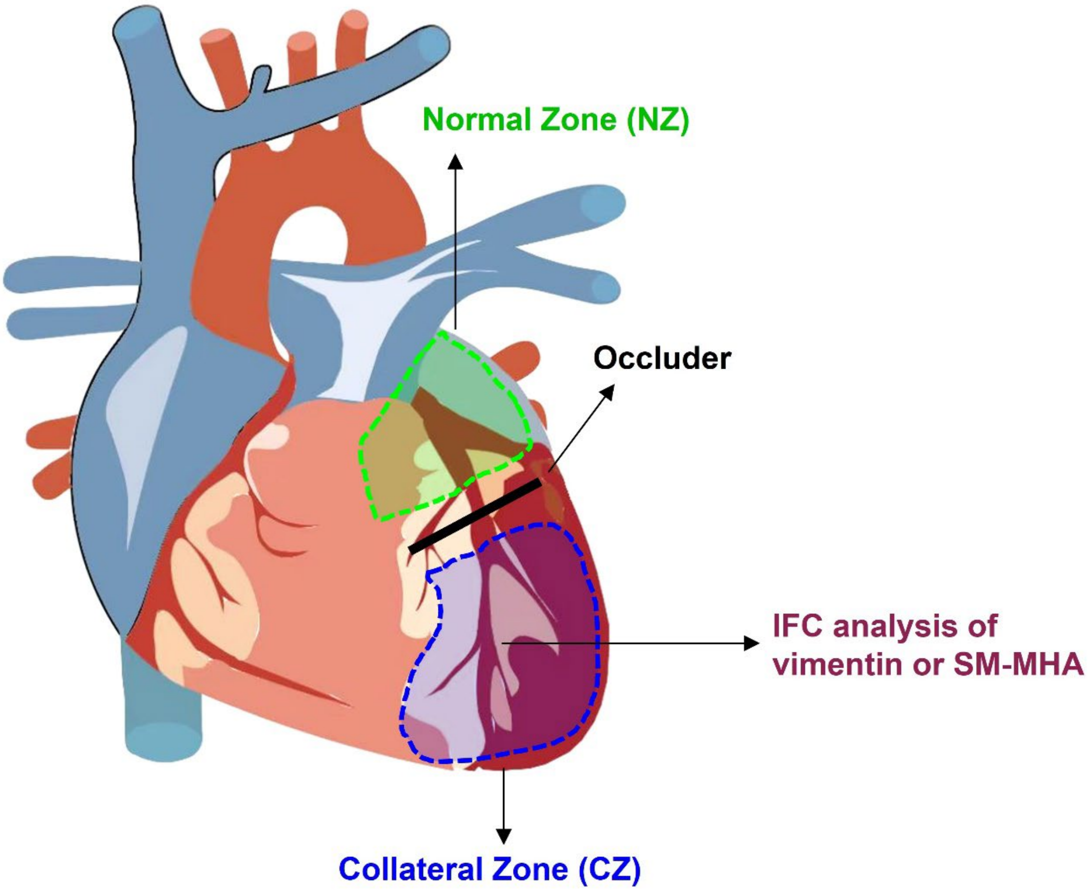

Appendix Figure S13. Schematic showing surgical placement of pneumatic snare around LAD artery for RI protocol. Normal zone (NZ) denotes the area above the pneumatic snare and collateral-dependent zone (CZ), the area below the pneumatic snare (ischemic during snare inflation). During the occlusion of LAD, CZ blood flow is dependent on the degree of coronary collaterals. The LAD tissue under the occulder was subjected to perform IFC analysis of vimentin and SM-MHC.

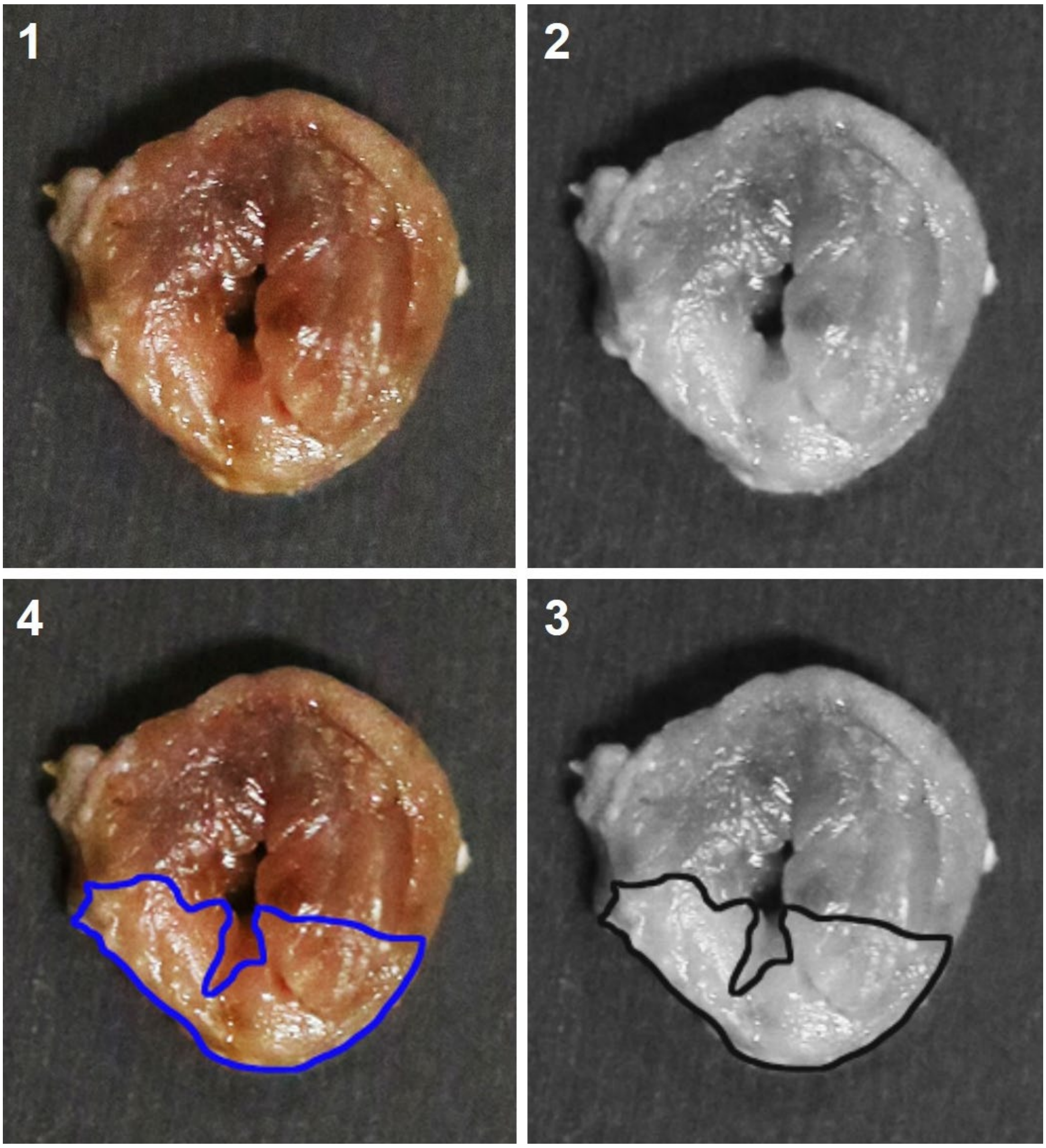

Appendix Figure S14. The quantitative method of infarct size using TTC staining. Step 1, hearts were subjected to perform TTC staining. The pictures were taken and then analyzed by Photoshop software. Step 2, the model of image was changed from color to gray (white and black). In gray-scale image, the white zone was defined as the infarct size. Step 3, the white zone was circled using a line and the area of the circled zone was calculated by the software automatically. The ratio of the circled area to the total area was defined as infarct size and expressed as (%). Step 4, the original picture was recovered by changing the model from gray to color. The circled line was labeled using blue.

Appendix Figure S15. Primers used in this study

Primer	Sequences
SM-MHC	forward, 5’-ATTGTCTTCCAAGAGTTCCGG-3’<br>reverse, 5’-TTGCTCTGCCCAATCCTG-3’
Vimentin	forward, 5’-TCCAGACTGTTGACCTCTTTG-3’<br>reverse, 5’-TCTTATGCTCCTGGGCTTTC-3’
p27	forward, 5’-AACTCCAGCTGGTCCTTAG-3’<br>reverse, 5’-TCTTGAACCCTCATC CTGT-3’
p21	forward, 5’-CGCGACAAGGCCAAGAT-3’<br>reverse, 5’-GCTGCTCCACCTTCTTCTG-3’
GADPH	forward, 5’-AGCTAAGAGAAGGGCGGAAC-3’<br>reverse, 5’-CATCTGCAGGCTGACATTGA-3’

Appendix Table S1. Effects of nitroglycerin (NTG) on *ex vivo* cardiac hydrokinetics parameters in rats following RI/MI

Groups	Vehicle		NTG		NTG+NAC	
	RI	RI+MI	RI	RI+MI	RI	RI+MI
N	15	12	10	11	15	13
Heart rate (beats/min)	302±39	439±45	327±33	428±35	313±40	497±49
LVDP (mmHg)	125±18	73±11[a]	113±17	52±16[b]	115±14	70±15[c]
+dP/dt_{max} (mmHg/s)	2916±237	1478±207[a]	2821±303	1007±319[b]	2891±199	1343±254[c]
-dP/dt_{max} (mmHg/s)	2236±158	1074±169[a]	2359±309	791±175[b]	2176±162	983±121[c]
Coronary flow (ml/min)	13.5±1.6	6.7±0.9[a]	12.9±1.7	4.2±0.7[b]	14.2±2.3	6.4±1.8[c]
Cholesterol (mM)	2.2±0.9	2.1±0.8	2.2±0.7	1.9±0.6	2.0±0.8	2.1±0.9
Triglyceride (mM)	0.97±0.30	0.92±0.27	0.93±0.21	0.89±0.26	0.87±0.25	0.99±0.28
Fasting glucose (mM)	5.2±1.7	5.7±2.9	5.9±1.0	5.0±1.2	4.9±0.8	5.3±1.9
Homocysteine (µM)	7.1±1.2	6.9±1.0	7.0±1.1	6.8±0.9	7.3±1.3	7.8±1.2

The protocols and experimental designs were described in Appendix Fig S1a. Data are expressed as means ± SEM. [a]$P < 0.05$ *vs.* Vehicle plus RI. [b]$P < 0.05$ *vs.* Vehicle plus RI+MI. [c]$P < 0.05$ *vs.* NTG plus RI+MI. A one-way ANOVA followed by Tukey *post-hoc* tests was used to determine *P* value in this table. *Ex vivo* measures were obtained in rats under normoxic conditions. LVDP, left ventricle developed pressure; +dP/dt_{max}, maximum rate of ventricular contraction; -dP/dt_{max}, maximum rate of ventricular relaxation.

Appendix Table S2. Effects of VSMC-specific iNOS gene deletion on *ex vivo* cardiac hydrokinetics parameters in *Apoe*$^{-/-}$ mice with hyperhomocysteinemia or hyperglycemia following RI/MI

Groups	*Apoe*$^{-/-}$			*Apoe*$^{-/-}$*/iNOS*$^{sm-/-}$		
	**Vehicle**	**HTL**	**STZ**	**Vehicle**	**HTL**	**STZ**
N	15	13	10	14	12	11
Heart rate (beats/min)	517±52	553±61	530±50	529±59	574±63	558±65
LVDP (mmHg)	61±18	38±13[a]	35±14[a]	67±11	58±7[b]	59±19[b]
+dP/dt_{max} (mmHg/s)	1784±129	1264±241[a]	1149±148[a]	1944±281	1882±301[b]	1839±313[b]
-dP/dt_{max} (mmHg/s)	1211±200	878±126[a]	812±153	1296±134	1250±141[b]	1191±127[b]
Coronary flow (ml/min)	5.7±0.5	3.3±0.4[a]	3.1±0.4[a]	5.8±0.6	5.2±0.5[b]	5.0±0.4[b]
Cholesterol (mM)	10.2±1.1	11.8±1.6	10.9±1.8	10.3±1.0	10.7±1.3	11.4±1.5
Triglyceride (mM)	0.87±0.21	0.93±0.17	1.05±0.13	0.86±0.14	0.95±0.12	0.87±0.19
Fasting glucose (mM)	5.5±1.5	4.7±1.2	10.8±3.2	5.1±1.1	6.2±1.3	11.7±2.2
Homocysteine (μM)	5.6±1.7	12.2±2.1	7.1±1.2	5.7±1.0	10.9±1.8	6.7±1.2
HTL (μM)	2.9±0.5	7.8±0.9	2.7±0.2	2.7±0.3	8.1±0.7	2.9±0.2

The protocols and experimental designs were described in Appendix Fig S7a. Data are expressed as means ± SE. [a]$P < 0.05$ *vs.* *Apoe*$^{-/-}$ plus Vehicle. [b]$P < 0.05$ *vs.* *Apoe*$^{-/-}$ plus HTL or STZ. A one-way ANOVA followed by Tukey *post-hoc* tests was used to determine *P* value in this table. *Ex vivo* measures were obtained in mice under normoxic conditions. LVDP, left ventricle developed pressure; +dP/dt_{max}, maximum rate of ventricular contraction; -dP/dt_{max}, maximum rate of ventricular relaxation.

Appendix Table S3. Effects of S-nitrosylation-resistant AMPKγ1 on *ex vivo* cardiac hydrokinetics parameters in *Apoe*$^{-/-}$ mice with hyperhomocysteinemia or hyperglycemia following RI/MI

Groups	WT-AMPKγ1			MT-AMPKγ1 (C130A)		
	Vehicle	HTL	STZ	Vehicle	HTL	STZ
N	13	14	11	15	13	10
Heart rate (beats/min)	502±39	557±57	539±45	513±46	527±49	519±51
LVDP (mmHg)	73±28	49±18[a]	43±11[a]	75±24	69±17[b]	64±15[b]
$+dP/dt_{max}$ (mmHg/s)	1809±175	1238±207[a]	1178±213[a]	1791±209	1743±180[b]	1687±154[b]
$-dP/dt_{max}$ (mmHg/s)	1136±158	896±109[a]	874±117[a]	1176±162	1107±139[b]	1074±111[b]
Coronary flow (ml/min)	5.1±0.4	3.2±0.5[a]	3.0±0.4[a]	5.6±0.6	5.3±0.4[b]	5.1±0.5[b]
Cholesterol (mM)	12.2±1.3	12.8±1.7	11.9±1.7	11.3±1.5	12.7±1.8	13.4±1.9
Triglyceride (mM)	0.83±0.20	0.91±0.16	1.03±0.11	0.89±0.12	0.91±0.10	0.88±0.17
Fasting glucose (mM)	4.5±1.0	4.8±1.4	11.8±2.2	4.1±1.0	5.2±1.7	13.7±2.9
Homocysteine (μM)	5.3±1.6	11.2±2.8	7.8±1.5	5.9±1.6	11.9±1.7	6.9±1.7
HTL (μM)	3.2±0.6	8.8±0.6	2.9±0.4	3.7±0.4	9.2±0.6	3.9±0.6

The protocols and experimental designs were described in Appendix Fig S9a. Data are expressed as means ± SE. [a]$P < 0.05$ *vs.* WT-AMPKγ1 plus Vehicle. [b]$P < 0.05$ *vs.* WT-AMPKγ1 plus HTL or STZ. A one-way ANOVA followed by Tukey *post-hoc* tests was used to determine *P* value in this table. *Ex vivo* measures were obtained in mice under normoxic conditions. LVDP, left ventricle developed pressure; $+dP/dt_{max}$, maximum rate of ventricular contraction; $-dP/dt_{max}$, maximum rate of ventricular relaxation.

Appendix Table S4. Demographic data for patients with acute MI

	Non	HHcy	Diabetes
Patient numbers (N)	100	100	100
Male (n)	67	63	69
Age (years old)	61±10	68±17	62±12
Body mass index (kg/m^2)	24.5±3.5	23.9±2.8	27.9±2.5
Systolic blood pressure (mmHg)	135±22	133±19	131±29
Diastolic blood pressure (mmHg)	77±11	72±13	71±9
Heart rate (bpm)	80±13	76±17	79±17
Fasting plasma glucose (mM)	5.1±1.9	6.8±1.4	8.9±2.7
Homocysteine (μM)	5.6±1.8	12.5±2.3	6.7±1.7
Triglyceride (mM)	1.8±1.1	2.3±1.2	2.1±1.0
Total cholesterol (mM)	4.3±1.2	4.8±1.6	5.1±1.9
High-density lipoprotein (mM)	1.0±0.2	1.1±0.2	1.3±0.2
Low-density lipoprotein (mM)	2.6±0.9	3.1±1.2	3.4±1.5
Smoking (n)	10	12	13
Drinking (n)	7	9	6
Hypertension (n)	16	14	19
